# All-dielectric metasurface for high-performance structural color

**DOI:** 10.1038/s41467-020-15773-0

**Published:** 2020-04-20

**Authors:** Wenhong Yang, Shumin Xiao, Qinghai Song, Yilin Liu, Yunkai Wu, Shuai Wang, Jie Yu, Jiecai Han, Din-Ping Tsai

**Affiliations:** 1 Ministry of Industry and Information Technology Key Lab of Micro-Nano Optoelectronic Information System, Harbin Institute of Technology, Shenzhen, Shenzhen, 518055 China; 2 Collaborative Innovation Center of Extreme Optics, Shanxi University, Taiyuan, 030006 China; 3 National Key Laboratory of Science and Technology on Advanced Composites in Special Environments, Harbin Institute of Technology, Harbin, 150080 China; 4 Department of Electronic and Information Engineering, The Hong Kong Polytechnic University, Hung Hom, Hong Kong

**Keywords:** Nanophotonics and plasmonics, Displays, Metamaterials

## Abstract

The achievement of structural color has shown advantages in large-gamut, high-saturation, high-brightness, and high-resolution. While a large number of plasmonic/dielectric nanostructures have been developed for structural color, the previous approaches fail to match all the above criterion simultaneously. Herein we utilize the Si metasurface to demonstrate an all-in-one solution for structural color. Due to the intrinsic material loss, the conventional Si metasurfaces only have a broadband reflection and a small gamut of 78% of sRGB. Once they are combined with a refractive index matching layer, the reflection bandwidth and the background reflection are both reduced, improving the brightness and the color purity significantly. Consequently, the experimentally demonstrated gamut has been increased to around 181.8% of sRGB, 135.6% of Adobe RGB, and 97.2% of Rec.2020. Meanwhile, high refractive index of silicon preserves the distinct color in a pixel with 2 × 2 array of nanodisks, giving a diffraction-limit resolution.

## Abstract (web summary)

Here, the authors fabricate a metasurface with high brightness and large gamut structured colors by combining a silicon metasurface with a refractive index matching layer. The experimentally demonstrated gamut is 181.8% of sRGB, 135.6% of Adobe RGB, and 97.2% of Rec.2020.

## Introduction

Colors, arising from the light-matter interaction, play vitally important roles in the world in which we live^1,2^. The most frequently used colors are produced by pigments and dye. Such colors are formed by absorbing certain wavelength range in visible^3^. They are typically dim and has a tiny gamut. The spot sizes of pigments are on the order of 25 µm, resulting in a poor resolution below 1000 dpi. To tackle the obstacles of pigments, the colors from structured materials have been revisited and several technologies are developed. One prominent example is the plasmonic structural color^4–7^. The interplay between light and plasmonic nanostructures such as gratings^8,9^, nanogaps^10,11^, and nanoparticles^12,13^ can produce vivid colors covering the entire visible spectrum. The local surface plasmon of individual Mie scatter has even pushed the imaging resolution to the resolution-limit of a bright-field microscope^14–16^. All-dielectric structural color is another important approach^17–19^. The electric/magnetic dipole modes and the collection resonance make the metasurfaces selectively transmit or reflect particular wavelengths. As some dielectrics are transparent in the visible, all-dielectric structural color are usually more vibrant and their gamut can be several times larger^20^. Recently, dynamically reconfigurable structural color have also been demonstrated by applying liquid crystals, microfluids, phase transition materials, or gain materials^21–23^.

**Fig. 1:**
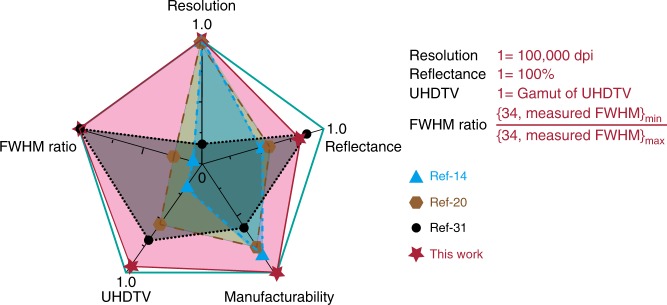
The direct comparison of different structural color technologies in spatial resolution, manufacturability, reflectance (or transmittance), FWHM ratio, and gamut. The triangles, hexagons, and circles represent the data taken from the ref. ^14^, ref. ^20^ and ref. ^31^. The stars correspond to the experimental results in this work. The value 34 in FWHM ratio is taken from ref. ^31^ and this work.

Despite of the above continuous successes, the practical applications of structural color in surface decoration^24^, digital displays^25^, molecules sensing^26^, optical security^27^, and information storage^28^ are still restricted. This is because both types of structural color only possess one or a few the above unique characteristics, far from the commercial requirements. This predicament is more overt in Fig. 1, where the recent breakthroughs are summarized using five key parameters—the manufacturability, the reflectance, the full width at half maximum (FWHM) ratio, the spatial resolution, and the gamut area in the international commission on illumination (CIE) color diagram. The plasmonic colors have subwavelength resolution but suffer from the low intensity and small gamut (~45% of sRGB)^29^. All-dielectric nanostructures such as TiO_2_ metasurfaces are distinct and vibrancy in bright-field^30^. But their spatial resolutions are an order of magnitude lower than their plasmonic counterparts. In addition, the record gamut area in experiment is only 68% of the Rec.2020^31^, far below the industrial requirements. Up to now, there is no structural color that can match all the above critical criterion simultaneously. Herein, we propose and experimentally demonstrate an all-in-one solution for structural color. By combing Si metasurface with a refractive index matching layer, high-brightness and distinct structural color with diffraction-limit resolution has been realized. The corresponding gamut area has been increased to 181.5% of sRGB.

## Results and discussions

**Fig. 2:**
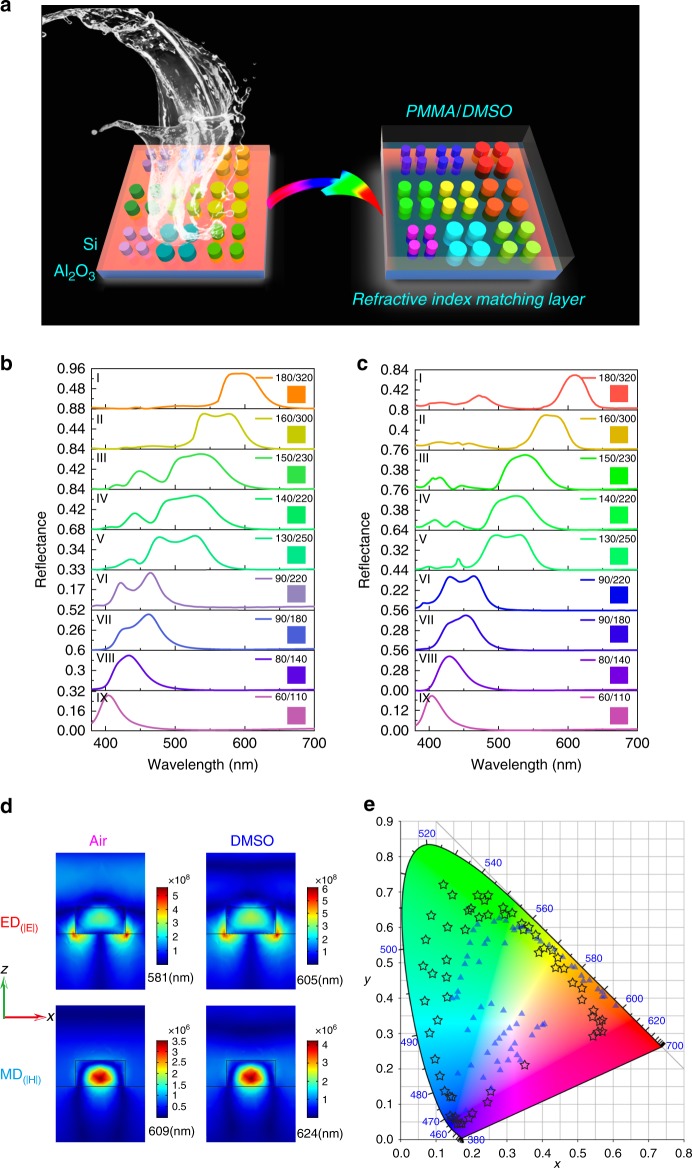
The schematic and the numerical simulation of Si metasurfaces. **a** The schematic of the Si metasurfaces. The left panel shows the colors are pale with a background reflection in air. The right panel shows distinct colors can be achieved by adding a refractive index matching layer such as PMMA or DMSO. Panel **b**, **c** are the calculated reflection spectra of Si metasurface with different lattice sizes in air and in DMSO, respectively. The insets are the corresponding structural color. From top to bottom, the panels are labeled as I–IX. **d** The electromagnetic field distributions of electric dipole mode and magnetic dipole mode in air and in refractive index matching layer with *n* = 1.48. Here the size parameter is diameter/period of 180/320 nm. **e** The CIE1931 plot for numerically calculated structural color palettes under Black Body illumination condition. The triangles and stars represent the results in air and in DMSO solution.

To achieve an ultimate solution, we revisited the Si metasurfaces. From the material side, silicon is extremely stable and compatible to the modern CMOS technologies, naturally matching the requirements on mass-manufacturability and long-time durability^18–20,32–34^. The sophisticated design on nanostructures gives the possibility of concealing its shortage and improving the color impression^20,35–37^. In addition, the large refractive index of Si enables simple structures for high-performance structural color, essential for the cost-effective nanofabrication^20,38,39^. Fig. 2a shows the schematic of the Si metasurface, which is composed of Si nanodisks on a sapphire substrate with radius *R* and lattice size *l*. The thickness is fixed at *h* = 100 nm. Panel I in Fig. 2b shows the numerically calculated reflection spectrum at the metasurface with diameter of nanodisk /lattice size of 180 nm/320 nm. Two resonances can be clearly seen at 581 and 609 nm, corresponding to the electric dipole (ED) resonance and magnetic dipole (MD) resonance (see left column in Fig. 2d), respectively. Due to the small thickness, the intrinsic material absorption is alleviated, and the maximal reflectance can be as high as 84%. As a result, orange color shall be clearly seen under a bright-field microscope (see inset in Fig. 2b). With the reduction of lattice size and the diameter of nanodisk, the reflection peak blue-shifts continuously, simply covering the entire visible spectrum. While the reflectance decreases with the wavelength, it is still about 84% at 600 nm and 29% at 402 nm, producing China pink, blue-violet, blue-gray, african-violet, caribbean-green, apple-green, bitter-lime, Erin and orange as well (panels I–IX and their insets in Fig. 2b). However, the displayed colors in Fig. 2b are still relatively pale. It can be seen more clearly when the structural color of 63 samples are plotted in the CIE1931 color map (triangles Fig. 2e). The gamut area is only 92% of sRGB, slightly larger than the gamut of plasmonic colors^29^.

The small gamut of Si metasurfaces is caused by the reflection background at the undesirable wavelengths and the broad reflection peak (see Fig. 2b and details in Supplementary Note 18). The first one produces a white background, whereas the second one spoils the color impression. To solve these problems, a refractive index matching layer is applied. Basically, the reduced refractive index contrast between sapphire and the refractive index matching layer can effectively suppress the reflection from the substrate. Similar to the situation to match the Kerker condition^20,40^, the destructive interference between electric dipole resonance and magnetic dipole resonance shall further decrease the undesirable reflection outside the main reflection peak. Meanwhile, the electrical dipole resonance is localized closer to the boundaries and is more sensitive to the environmental refractive index changes than the magnetic dipole. As a result, the refractive index matching layer can push the electric dipole resonance to the magnetic dipole resonance and thus narrow the FWHM of reflection spectrum. Figure 2c shows the reflection spectra of the same Si metasurfaces in Fig. 2b but coated with a refractive index matching layer with *n* = 1.48. As expected, both the white-background and the FWHM are well suppressed by the Kerker condition, which are caused by the reduced refractive index contrast and the different responses of electric and magnetic dipole resonances (see Supplementary Note 18). The reflectance of magnetic dipole resonance is well preserved at ~74%–21% in the visible spectrum. The modifications on background reflection and FWHM look trivial but are very important to pushing the performances of structural color to the limit, i.e. Rec.2020, a standard for ultrahigh-definition television since 2012. Consequently, more vivid structural color can be seen in the insets of Fig. 2c.

For a direct comparison, we have also plotted these colors as stars in the same CIE 1931 color map in Fig. 2e. With the refractive index matching layer, the simulated color gamut is around 186% of the sRGB, 138.7% of the Adobe RGB, and even approaches 99.5% of the Rec.2020. Similar to the conventional all-dielectric metasurfaces^20,30,41,42^, the structural color from Si metasurface with a refractive index matching layer (*n* = 1.48) are also independent to the incident angle (see Supplementary Note 19) and only slightly increase the material absorption (see Supplementary Note 20). Therefore, adding a refractive index matching layer to high refractive index all-dielectric metasurface is an effective approach to improve the structural color to the limit.

**Fig. 3:**
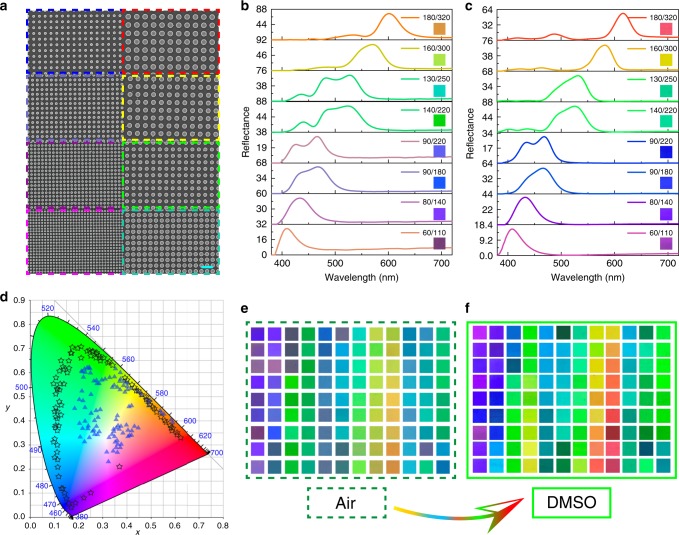
The experimentally recorded structural color. **a** the top-view SEM images of the Si metasurfaces with different lattice sizes. The scale bar is 400 nm. Panels **b**, **c** are the experimentally recorded reflection spectra in air and in DMSO solution. The insets are their displayed colors under bright-field microscope. **d** The experimentally recorded color palettes of 108 Si metasurfaces in air (triangles) and in DMSO (stars), respectively. Panels **e**, **f** are the experimentally recorded color palettes of 108 Si metasurfaces in air and in DMSO, respectively.

We then fabricated the designed nanostructures on a commercially available silicon on sapphire (SOS) wafer (http://www.roditi.com/SingleCrystal/silicon-on-sapphire.html) with a combined process of electron-beam lithography and reactive ion etching (see the “Methods” section). To test the influences of refractive index matching layer, the metasurfaces were integrated into a microfluidic channel after the fabrication and simply infiltrated with dimethyl sulfoxide (DMSO) with *n* = 1.48. The color performances were optically characterized with a bright-field optical microscope (see the “Methods” section). Figure 3a shows the top-view scanning electron microscope (SEM) images of the Si metasurfaces with different lattice sizes. Figure 3b, c summarize their reflection spectra. High reflection peaks appear at 406, 433, 472, 467, 526, 529, 576  and 600 nm when the metasurfaces are placed in air. China pink, blue-violet, blue-gray, african-violet, caribbean-green, apple-green, Erin and orange are also recorded under a bright-field microscope. Once the DMSO is infiltrated as the refractive index matching layer, the reflection peaks shift (see Fig. 3c) and the structural color immediately transit to Fandango, electric violet, dark violet, blue, cyan, bright green, yellow, and red (insets in Fig. 3c), demonstrating the possibility in dynamic color displays. More than the wavelength shift, the FWHMs of main peaks are around 34–40 nm in Fig. 3c, close to the FWHM of TiO_2_ metasurfaces^30,43^ and 40% narrower than the ones in air (see Fig. 3b). The background reflection from substrate is also suppressed to ~zero (see Supplementary Fig. 2). As a result, the 108 structural color in Fig. 3f become more distinct and vibrancy than the ones in air (Fig. 3e). By plotting the structural color in the same CIE 1931 colormap (see Fig. 3d), we can see that the gamut area has been increased from 78% of sRGB in air to around 181.8% of sRGB, 135.6% of Adobe RGB, and 97.2% of Rec.2020 in DMSO.

The above results are different from the previous reports on hybrid nanostructures. In previous reports^31^, while the simulated gamut can also be around 171% of sRGB, the experimentally demonstrated gamut is only about 124% of sRGB (68% of Rec.2020). This is because the fabrication technologies of different materials are in-compatible and the top layers are usually deformed during the etching of bottom materials. In current experiment, only single-crystalline silicon has been employed in the nanofabrication process. The CMOS compatible fabrication technology of Si is mature and highly reproducible. As a result, the nanostructures can be well produced by lithography and transferred to silicon via reactive ion etching. The high-resolution tilt-angle SEM images show that the roughness is smaller than 10 nm and the sidewall is nearly 90° in the vertical direction (Supplementary Fig. 4). Therefore, both of reflectance and the gamut area perfectly follow the design in Fig. 2 and a record large gamut area of structural color has been experimentally realized. Meanwhile, the single-crystalline Si has lower material absorption than poly-crystalline Si and amorphous Si in the visible spectrum, making the gamut area in Fig. 3d 110 and 238% larger than the latter two materials.

**Fig. 4:**
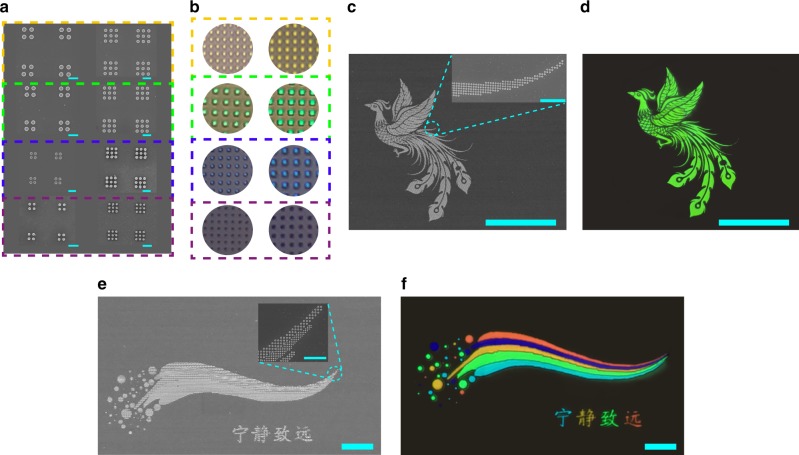
The resolution test of the all-dielectric structural color. **a** The top-view SEM images of Si metasurfaces with different lattice and different pixel sizes. The scale bar is 400 nm. **b** The experimentally recorded bright-field microscope images. The smallest pixel sizes for yellow, green, blue, and purple colors are 320, 250, 200, and 190 nm, respectively. **c** The top-view SEM images of “Phoenix”. **d** The pure color image of a “Phoenix” with different pitch sizes. No color degradation can be seen with the reduction of pixel size. The insets show the SEM images at different locations. **e** The top-view SEM images of “rainbow”. **f** The microscope image of a color “Rainbow” with different pixel sizes. Different colors can be well distinguished at the diffraction-limit resolutions. The scale bars are 100 µm in (**c**, **d**), and 20 µm in (**e**, **f**). The scale bars of insets in (**c**, **e**) are 250 nm.

In addition to the high reflectance, small FWHM, large gamut, mass-manufacturability, and long-time durability, the spatial resolution is another key parameter for structural color. In TiO_2_ nanostructures, the dependence on collective resonance restricts the resolution around 10^4^ dpi^30^. Here the high refractive index of Si has the potential to improve the spatial resolution. To test the resolution, we have fabricated a series of samples consists of 3 × 3 and 2 × 2 arrays of Si disks per pixel (see Fig. 4a). The center-to-center distances for yellow, green, blue, and purple colors are 320, 250, 200, and 190 nm, which correspond to the resolution limit of the optical microscope (×100, NA = 0.9) at their operating wavelengths. Figure 4b summarizes the corresponding bright-field structural color. While the number of disks per pixel has been reduced to nine and four, the colors of each pixel is still well preserved. Compared with the simple reduction of pixel size, it is more essential to ensure that the color impression and color distinction will not be spoiled. These are the fundamental bases for the construction of high-resolution color image. To test the first characteristic, we have fabricated pure color “Phoenix” patterns with different pixel sizes (see the other patterns in Supplementary Note 3–9). While the pixels of “Phoenix” vary from 5 × 5 to 2 × 2 (see SEM images in Fig. 4c), the Phoenix patterns are quite uniform both in air (see Supplementary Fig. 3) and in DMSO solution (Fig. 4d), even with a large viewing angle (see Supplementary Fig. 22). These images show that the color impression produced by Si metasurfaces will not degrade obviously with the decrease of pixel sizes. Figure 4e and f show another “Rainbow” pattern consisted of different structural color. From left to right, the pixel sizes keep decreasing from 5 × 5 to 2 × 2 array of Si nanodisks. The microscope images show that different colors can still be distinguished even they approach the diffraction limit. The small pixel size, color uniformity, and color distinguishability in Fig. 4 have confirmed that Si metasurfaces can produce structural color with diffraction-limit resolution.

**Fig. 5:**
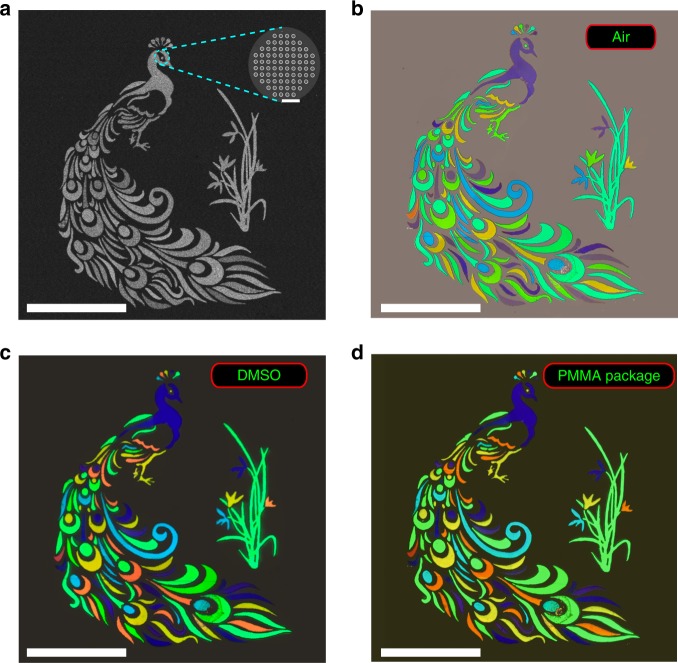
Full-color image printing with Si metasurface. a The top-view SEM image of a peacock with an orchid. The scale bar is 100 µm. Panels **b**–**d** are the corresponding bright-field microscope images in air, DMSO solution and packaged with PMMA. The scale bar is 100 µm, and the scale bar of inset SEM is 1 µm.

At last, we have presented a microscopic color image of a “Peacock” to demonstrate the creation of arbitrary images. Figure 5a shows the top-view SEM image of the peacock. The overall size of the image is 220 × 260 µm. The colors of the “Peacock” are defined by controlling the lattices size *l* and radius *R*. Owing to the preserved color impression and distinction in Fig. 4, the pixel sizes vary between 9 × 9 and 2 × 2 array of Si nanodisks to fit the entire image. When the sample is illuminated with a white light source under bright-field microscope, a “Peacock” image with uniform and distinguished green, blue, and purple colors can be clearly seen in Fig. 5b. After the infiltration of DMSO solution, the “Peacock” changes immediately and the new image consists of red, green, and blue colors (see Fig. 5c). By comparing Fig. 5b and c, it is easy to see that the color image can be dynamically switched. Meanwhile, the background in DMSO solution is almost completely dark. Similar to all the above studies, the “Peacock” image becomes more distinct and vibrancy. This is also a direct proof of the reduction of background reflection in Figs. 2 and 3. Note that the refractive index matching layer is not limited to the solution such as DMSO. It is also applicable to liquid crystals^44^ or solid-state materials (see Supplementary Note 21). Figure 5d shows the image of the “Peacock” after packaging with polymethyl methacrylate (PMMA). It is almost the same as the result in Fig. 5c.

In summary, we have proposed and experimentally demonstrated structural color produced by the Si metasurfaces. By applying an index matching layer, the structural color from Si metasurfaces can possess a series of unique properties, i.e. high reflectance (76% at 600 nm), narrow FWHM (~34 nm), negligible background reflection. As a result, the gamut area has been pushed to a record value around 97.2% of Rec.2020. and the spatial resolution is increased to the diffraction limit without spoiling the color uniformity and impression. As the nanostructures are purely made of silicon, they naturally inherit the mass-manufacturability and long-time stability characteristics of silicon photonics. As the stars plotted in Fig. 1, it is clear to see that all the critical criterion for a good structural color has been realized in Si metasurfaces. This research routes a key step towards the commercialization of structural color in dynamic displays, optical security, and information storage as well (http://www.roditi.com/SingleCrystal/silicon-on-sapphire.html)^45^.

## Methods

### Numerical simulation

The simulations of the reflectance spectrum are calculated by the commercial software Lumerical FDTD Solutions and the COMSOL Multiphysics. The periodic condition is used in the plane to mimic the periodic structures. Perfectly matched layers are applied in the transmission and reflection directions to absorb the outgoing waves. The refractive index of single-crystalline silicon is taken from the material date of the software. The refractive index of sapphire is fixed at 1.76. A MATLAB code is used to calculate the xy coordinates, which are plotted in International Commission on Illumination (CIE) 1931 chromaticity diagram (see details in Supplementary Note 16). The contributions of electric dipole and magnetic dipole resonances are analyzed with the multipolar decomposition (see Supplementary Note 18).

### Fabrication and optical measurements

The Si metasurfaces are fabricated with electron beam lithography technique followed by a reactive ion etch (RIE) process in an Oxford Plasma System (Oxford 80). Basically, the inversed nano-pattern is generated within a PMMA electron beam resist (A2, Microchem). The pattern is transferred to Cr mask via a lift-off process. Then the Si nanostructures are produced by the RIE process and removing the Cr mask with the chromium etchant (see details in Supplementary Note 10).

The samples are placed onto an optical microscope (ZEISS, Axio Scope AI) stage associated with a home-made optical setup to control the polarization and incident angle. The reflection spectrum is recorded with a spectrometer under the ×50 objective lens (NA = 0.55), see details in Supplementary Note 11.

## Supplementary information

Supplementary Information

## Data Availability

The data that support the findings of this study are available from the corresponding author upon reasonable request.
