# Supplementary material for: All-dielectric metasurface for high-performance structural color
Source: Nat Commun. 2020 Apr 20;11:1864. doi: 10.1038/s41467-020-15773-0 (PMC7171068; doi:10.1038/s41467-020-15773-0)
Supplement: Supplementary file 1 — Supplementary Information [file 41467_2020_15773_MOESM1_ESM.pdf]

Supplementary Information for

All-Dielectric Metasurface for High-Performance Structural Color

Wenhong Yang[1], Shumin Xiao[1, 2, 3, #], Qinghai Song[1, 2], Yilin Liu[1], Yunkai Wu[1], Shuai Wang[1], Jie Yu[1], Jiecai Han[3], Din-Ping Tsai [4, *]

1. Ministry of Industry and Information Technology Key Lab of Micro-Nano Optoelectronic Information System, Harbin Institute of Technology, Shenzhen, Shenzhen, China, 518055.
2. Collaborative Innovation Center of Extreme Optics, Shanxi University, Taiyuan 030006 China.
3. National Key Laboratory of Science and Technology on Advanced Composites in Special Environments, Harbin Institute of Technology, Harbin 150080, China.
4. Department of Electronic and Information Engineering, The Hong Kong Polytechnic University, Hong Kong.

Corresponding authors: # shumin.xiao@hit.edu.cn; * dinping.tsai@polyu.edu.hk

Supplementary Text

Supplementary Note 1: The comparison of reflectance between metasurfaces in Air and DMSO

To clarify the suppression of substrate reflectance and broad resonance modes clearly by introducing the reflective index matching layer, we plot the simulated and experimental reflectance spectra of the sample with diameter/period (nm) equal to 110/260 nm in air and DMSO, respectively. As shown in Supplementary Figure 1(a)-(b) represent the reflectance for the sample with green color. The experiment and simulation results match very well to each other. The substrate reflectance is high in visible wavelength (380-700 nm), and the resonance modes are broad as well in the Air condition (Supplementary Figure 1(a)). After adding the refractive index matching layer (DMSO), the substrate reflectance can be suppressed almost to zero, and the full width of half-maximum (FWHM) can be decreased to 55 nm in the experiment results (Supplementary Figure 1(b)).

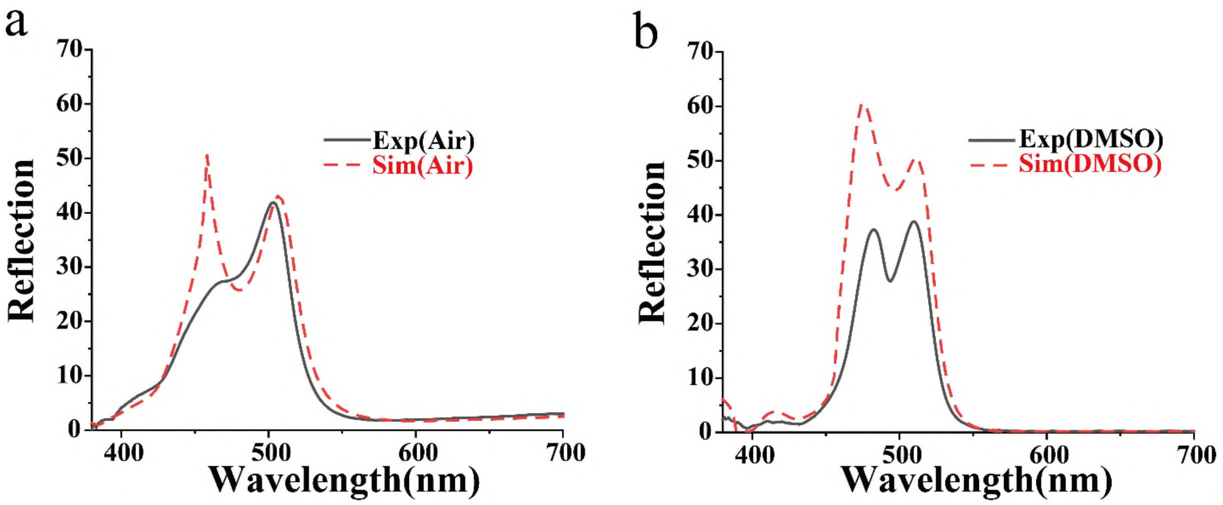

Supplementary Figure 1. (a)-(b) The reflectance spectra of a sample with diameter/period equal to 110/260 nm in Air and DMSO, respectively. The black solid line represents the experiment result and the dashed line represents the simulation result calculated by the FDTD Solutions.

Supplementary Note 2:The effect of gamut with the different environmental refractive index (n)

The small gamut of previous silicon metasurfaces is caused by the reflection background from the substrate as well as the broad resonant modes. With the increasing of the refractive index of the media surrounding the nanodisks from 1 to 1.5, the reflection background through the whole spectrum range becomes lower (see inset in Supplementary Figure 2(a)) and the broad resonant mode becomes narrower as shown in Supplementary Figure 2(a). When the refractive index increases to 1.5, the reflection from the substrate will be decreased as small as zero. The 1931 CIE (x, y) chromaticity coordinates are calculated from the spectra and plot on Supplementary Figure 2(b) with the six dots. The direction of the black arrow in Supplementary Figure 2(b) represents the increasing of the refractive index. When the environment media is air with n=1, the chromaticity coordinate almost locates in the middle of the CIE 1931 chromaticity diagram, which means the color is not pure and saturation is low. With the increase of the refractive index, the chromaticity coordinate of dot keeps moving towards the outside and more and more close to the boundary, indicating that the color becomes purer and more saturated.

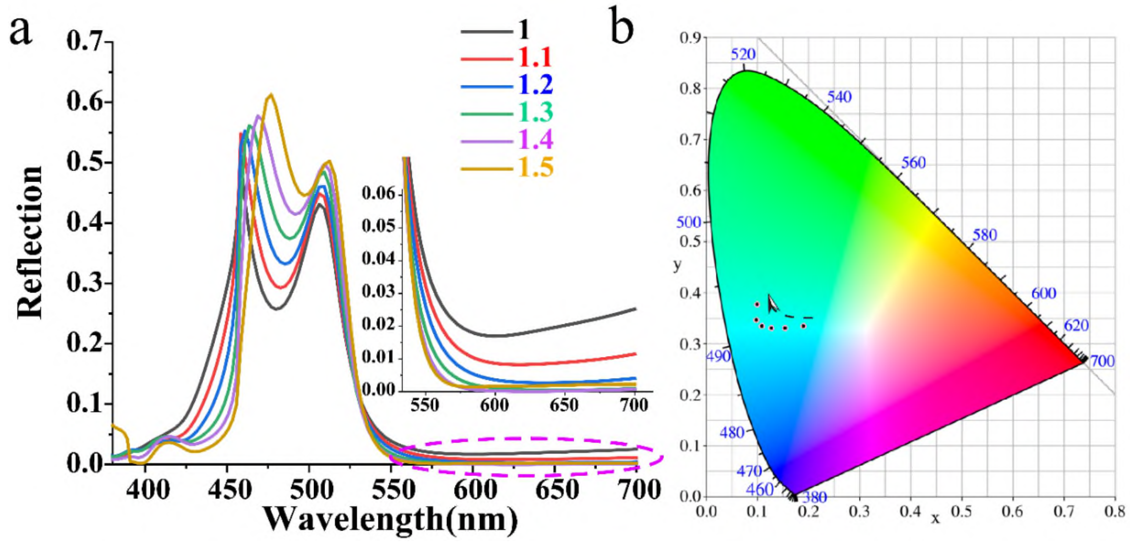

Supplementary Figure 2. (a) The simulative reflectance spectra of metasurface embedded in media with refractive indexes increasing from 1 to 1.5. The inset is the magnifying image of the area surrounded with the green circle. (b) The colors corresponding to different reflectance are plotted in the CIE 1931 chromaticity diagram. The direction of the arrow represents the increasing of the refractive index for the surrounding media.

Supplementary Note 3: The high-magnification optical microscope image of rainbow and green phoenix in air and DMSO

In the main text, we have discussed the colors of a rainbow and a phoenix in DMSO. Here, we demonstrate the original pictures and their structural color on air for a direct comparison. As illustrated in Supplementary Figure 3, both of the rainbow and phoenix are colorful in air but away from design. Their colors are relatively pale and the background reflection is strong. For a direct comparison, the images in DMSO are plotted again. It is easy to see the improvements with the infiltration of DMSO. These results are also consisting with Supplementary Figure 1 and Supplementary Figure 2 very well.

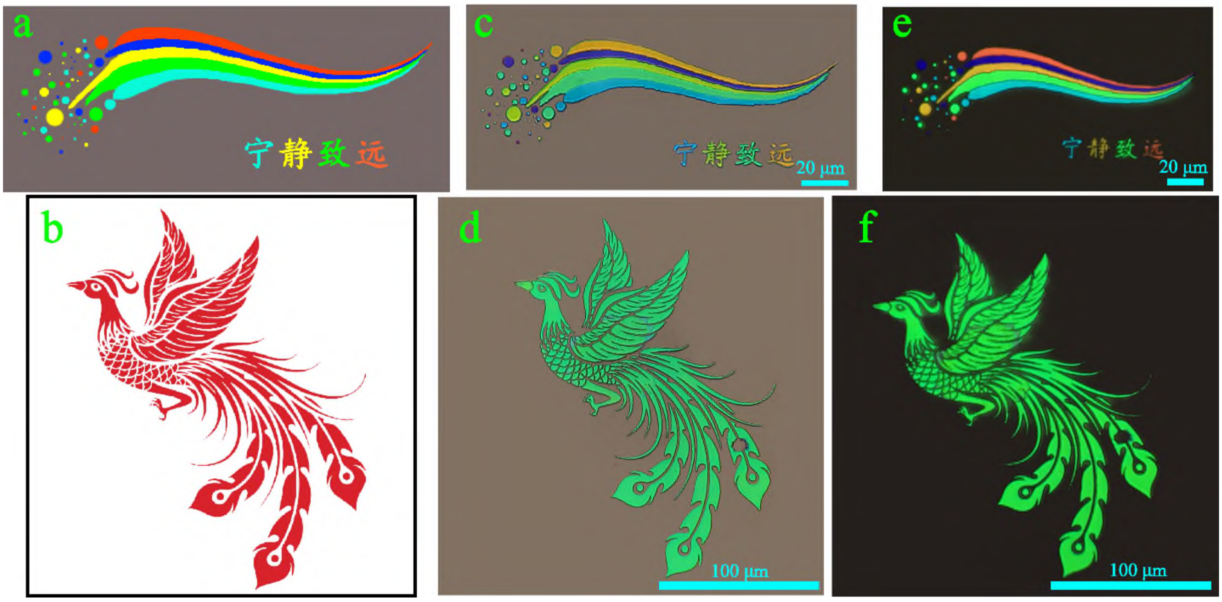

Supplementary Figure 3. (a) (b) The original design image of the rainbow and phoenix in the air. (c) (d) The optical microscope image of the pale rainbow and apple green phoenix in air. (e) (f) The optical microscope image of the colorful rainbow and bright green phoenix in DMSO. The scale bars are shown in SEM image.

Supplementary Note 4:The SEM images of different color in the rainbow

In our structural color, the displayed colors are controlled by the diameter of Si nanodisk and lattice size. Here we take the rainbow as an example to illustrate it. As depicted in Supplementary Figure 3(c) and Supplementary Figure 3(e), the rainbow has five different colors. The corresponding SEM images (top row for top-view SEM and the bottom row for side-view) are plotted in Supplementary Figure 4. It is clear to see that the structural parameters determine the colors in Supplementary Figure 3.

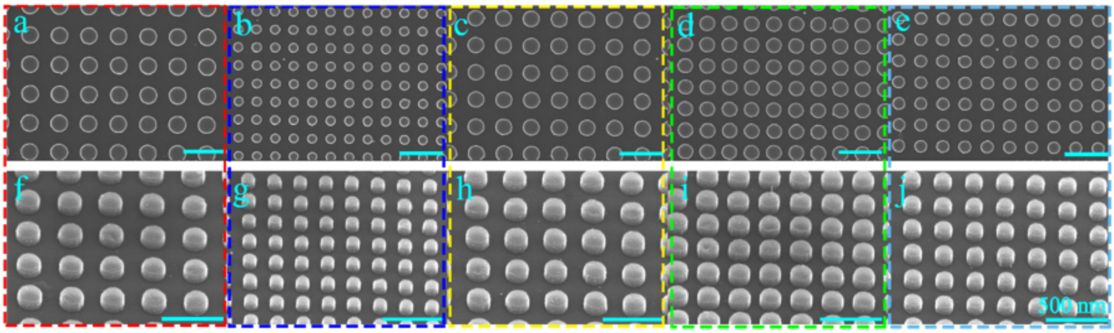

Supplementary Figure 4. (a)-(e) The top view SEM images of the red, blue, yellow, green and cyan colors in the rainbow optical microscope image. (f)-(j) The corresponding tilt-view SEM images of the different colors in (a). The diameter/period of these five colors are 170/320 nm, 90/200 nm, 160/300 nm, 150/240 nm, and 120/240 nm, respectively. The scale bar is 500 nm.

Supplementary Note 5: The other pure color of the phoenix

We also design the phoenix with other colors of dark orchid, dark cyan, Bitter lime and Carrot orange in air (Supplementary Figure 5(a)-(d)). The smallest pixel is 2 × 2 in each color. The maximum resolution reaches beyond 100,000 d.p.i. After the samples are immersed into the DMSO solution, the four colors change to blue, cyan, yellow and red, respectively (Supplementary Figure 5(e)-(h)). All of the four colors become purer with higher saturation.

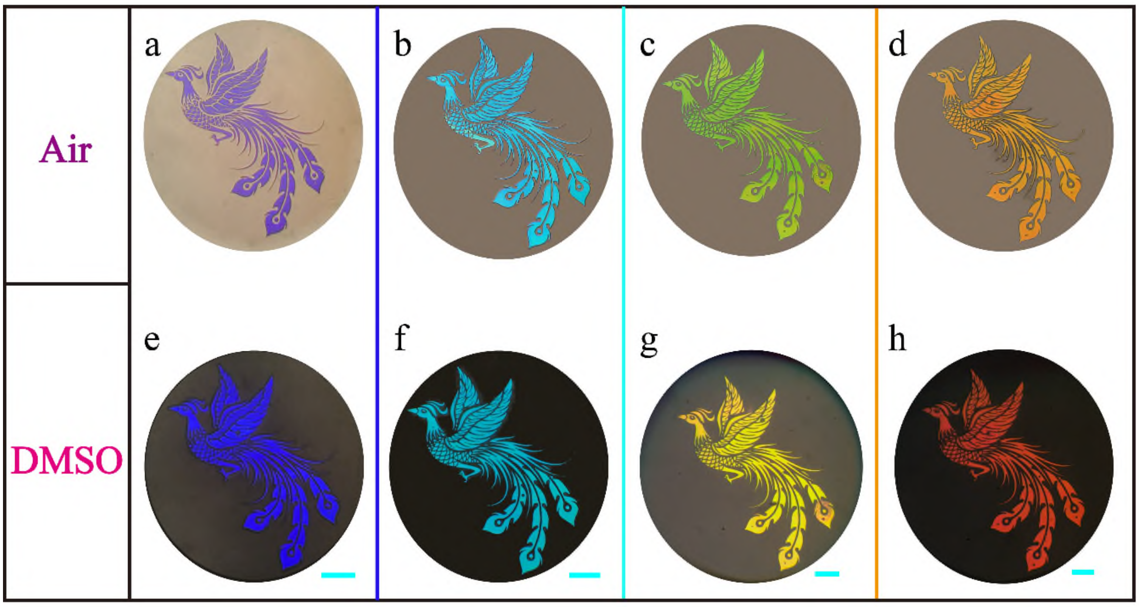

Supplementary Figure 5. (a) The optical microscope images of phoenix with four different colors in the air. (b) The optical microscope images of phoenix with four colors embedded in DMSO. The diameter/period (nm) of four colors are 90/200 nm, 120/240 nm, 160/300 nm, and 170/320 nm, respectively. The scale bars are 30 μm.

Supplementary Note 6:The pure color of the “福”

In China traditional culture, the Chinese character “福” represents blessing. Here, We design two kinds of Chinese paper cutting pattern“福” with plum blossom and two fish, respectively. When the two kinds of “福” in air, the dark orchid, dark cyan, Apple green, Bitter lime, and Carrot orange are presented in Supplementary Figure 6(c)-(g) and (m)-(q). After adding the refractive index matching layer DMSO on the two kinds of “福”, the color change to blue, cyan, bright green, yellow and red shown in Supplementary Figure 6(h)-(l) and (r)-(v), which become purer and saturation.

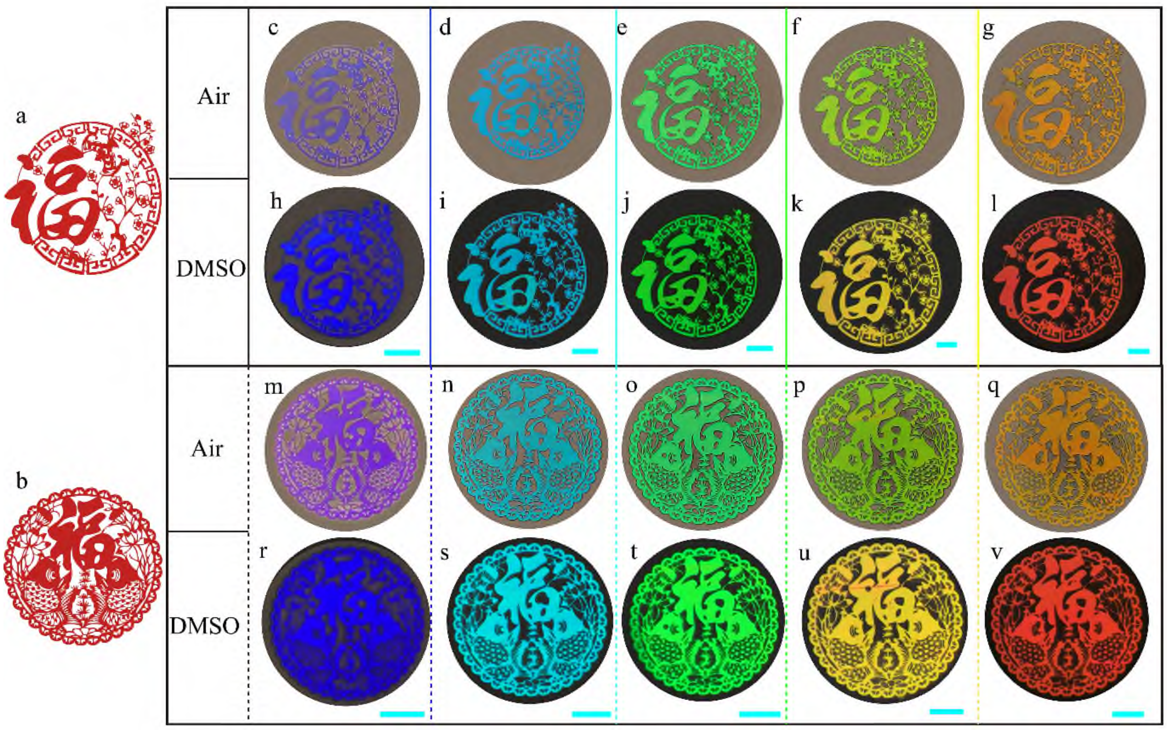

Supplementary Figure 6. (a) and (b) are the original design Chinese paper cutting pattern“福” with plum blossom and two fish. The first row (c)-(g) and The third row (m)-(q) the optical microscope image of “福” with five kinds of color in the air. The second row (h)-(l) and The fourth row (r)-(v) the optical microscope

image of **"福"** with five kinds of colors in DMSO. The diameter/period (nm) of five color are 90/200 nm, 120/240 nm, 150/240 nm, 160/300 nm and 170/320 nm, respectively. The scale bars are 30 μm.

Supplementary Note 7: The SEM image two kinds of “福”

We also characterize the sample of diameter/period (nm) 120/240 nm (Supplementary Figure 7(a)-(b)) and 160/300 nm (Supplementary Figure 7(c)-(d)) with SEM images. The smallest detail can be clearly distinguished in these patterns which are comprised of 2 × 2 silicon nanodisks, indicating that the maximum resolution can be reached beyond 100,000 d.p.i.

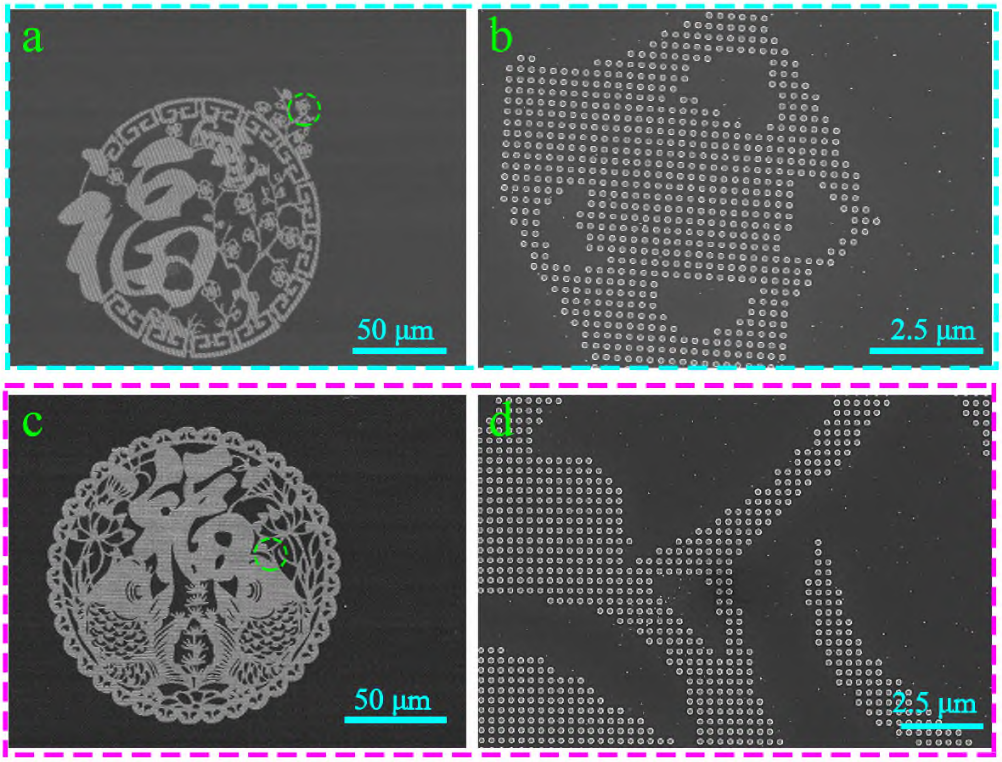

Supplementary Figure 7. (a) The SEM image of cyan **“福”** with plum blossom (b) The magnifying SEM image in (a) surrounded with the green circle (c) The SEM image of yellow **“福”** with two fish. (d) The magnifying SEM image in (b) surrounded with the green circle. The scale bars are shown in the SEM image.

Supplementary Note 8:Optical and scanning electron micrographs of checkerboard patterns

To show the highest resolution of silicon nanodisk. We design the chequerboard consists of two colors with different diameter and the same period. The one brighter than the other. The optical micrograph images for checkerboard with 2 × 2 and 3 × 3 silicon nanodisks are shown in Supplementary Figure 8(a)-(d), the two colors of chequerboard pattern can be distinguished with a ×100 and 0.9 NA objective, demonstrating the patterning of color pixels at the optical diffraction limit. Under identical illumination conditions, both 2 × 2 and 3 × 3 pixels of nanodisks exhibit similar color, the color only influenced by the dimension, not the array number. The maximum resolution of the design can be reached beyond 100,000 d.p.i.

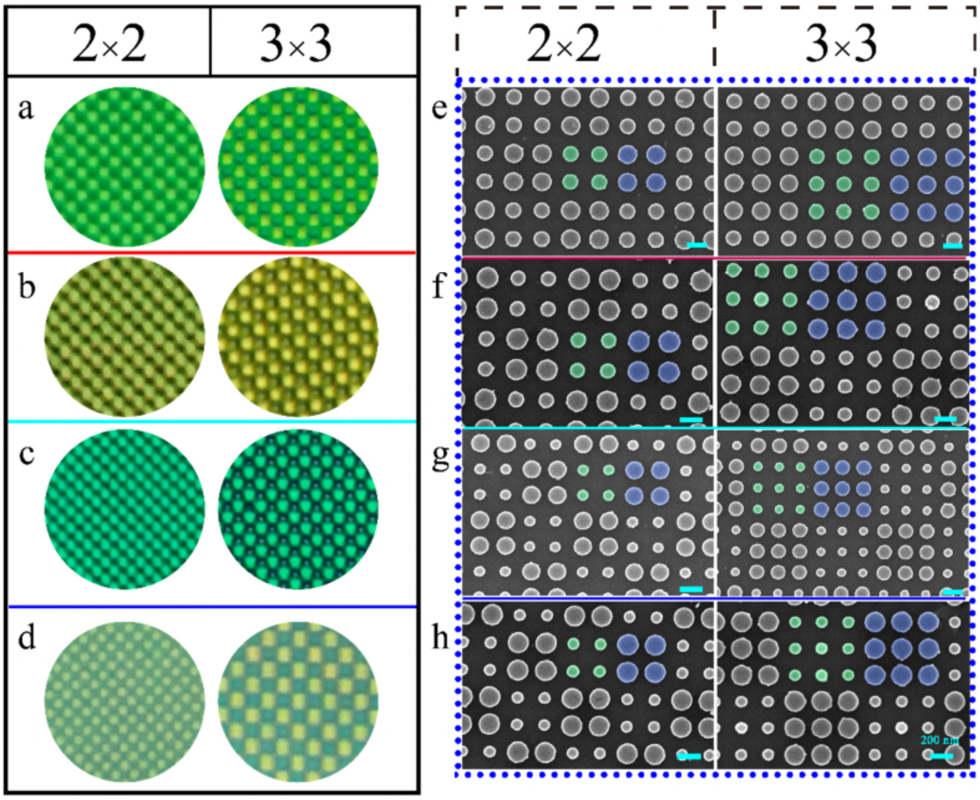

Supplementary Figure 8. (a)-(d) Optical micrograph images of the checkerboard with 2 × 2 and 3 × 3 silicon nanodisks. (e)-(h) the SEM image of checkerboard consisting of 2 × 2 and 3 × 3 nanodisks. The diameter/diameter /period (nm) of checkerboard from up to down are 170/140/280 nm, 170/120/270 nm, 140/80/220 nm, and 170/90/240 nm, respectively. The scale bars are 200 nm.

Supplementary Note 9: Optical and scanning electron micrographs of the abstract pattern and the name

Similar to the “Rainbow”, “Phoenix”, and “福”, we have also designed and fabricated many other nanostructures to illustrate the high resolution of the structural color. Supplementary Figure 9(a)-(c) show that the name of the first author in Chinese characters. The top-view SEM image that the figure consists of 2 × 2 and 3 × 3 nanodisk arrays. The structural color can be clearly seen in both air and DMSO, respectively. Supplementary Figure 9(d)-(g) show that the high-resolution colors can still be distinguished when they are distributed closely in a pattern.

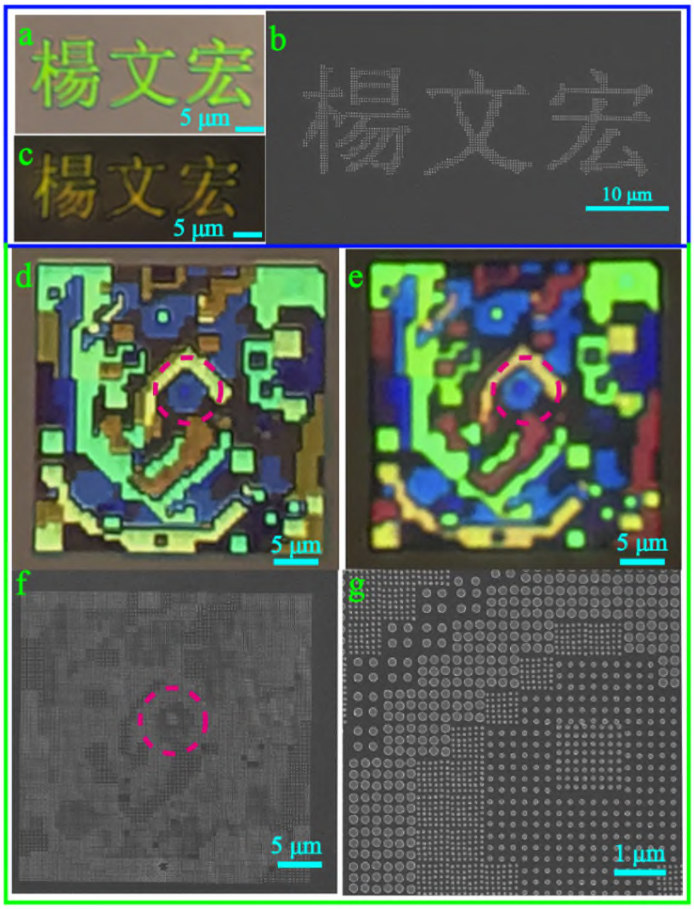

Supplementary Figure 9. (a)-(b) Optical micrograph images of the name in Air and DMSO, respectively. (c) The SEM image of the name. (d) Optical micrograph images of the pattern in Air and DMSO, respectively. (e) Optical micrograph images of (f) The SEM image of pattern (g) The magnifying SEM image in (f) surrounded

with the wine red circle. The 2 × 2 silicon nanodisks pixel can be clearly distinguished in SEM images. The scale bars are shown in the SEM image.

Supplementary Note 10: The fabrication process of the silicon nanodisk with PMMA photoresist

The metasurfaces were fabricated with electron beam lithography technique followed by a lift-off process. Firstly, we cleaned the 100 nm silicon sapphire substrates in the ultrasound bath in acetone and isopropyl alcohol (IPA) for 10 min, respectively. Secondly, 80 nm PMMA film was spin-coated onto the silicon-coated sapphire substrate and the substrate is baked at 180 °C for an hour. After that, the PMMA resist was exposed to the electron beam (Raith E-line, 30 kV) and developed in MIBK/IPA solution for 60 s at 0 °C to form the PMMA nanostructures. Then the sample was transferred into an E-beam evaporator and directly coated with 25 nm Cr films (deposition rate 0.5 Å/s, base vacuum pressure 5×10^{-7} Torr). After immersing the sample in acetone for 8 h, the PMMA was removed and the nanostructures were well transferred to Cr. Then the silicon was etched away with reactive ion etch (RIE) performed in an Oxford Plasma System using CHF_3 and SF_6 gases. Finally, by immersing the sample into the chromium etchant for 10 min, Si metasurfaces were finally obtained.

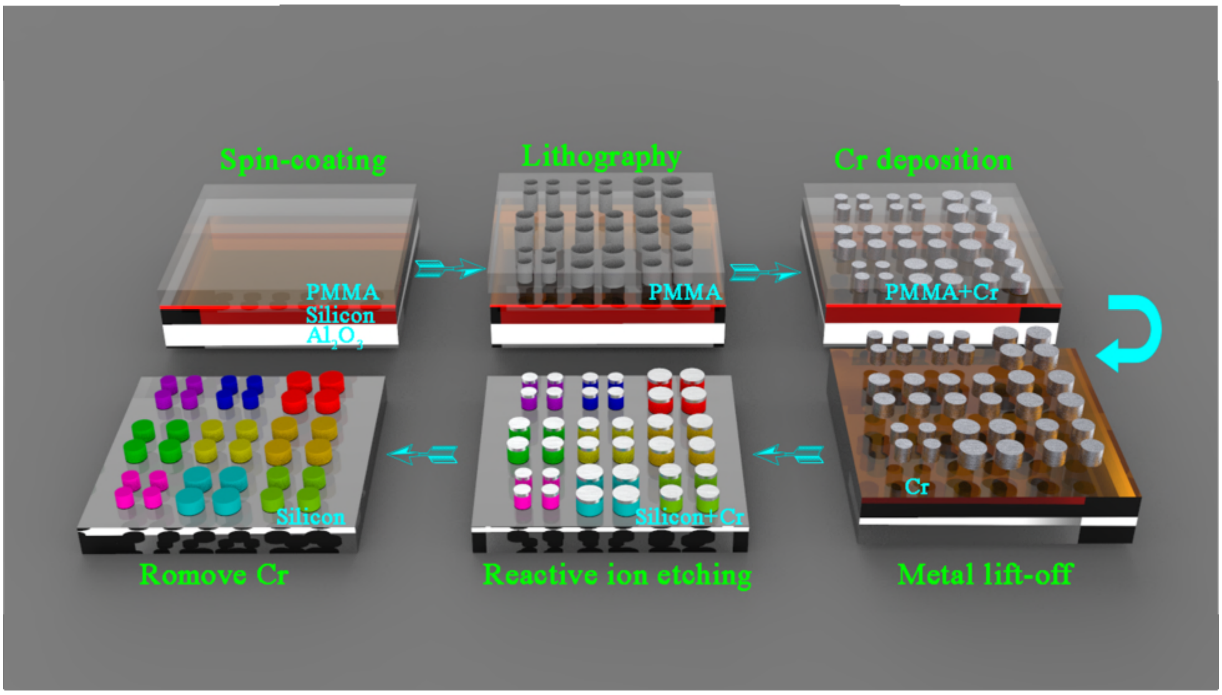

Supplementary Figure 10. The schematic of the fabrication process for silicon nanodisk based on PMMA photoresist.

Supplementary Note 11:The optical characterization progress of the structural color

The sample was placed onto an optical microscope (ZEISS, Axio Scope AI) stage under a homemade light path. White halogen lamp is illuminated normally onto the samples and the reflection spectrum were taken with a spectrometer. The bright-field microscopy images were taken using the same setup with iPhone 7.

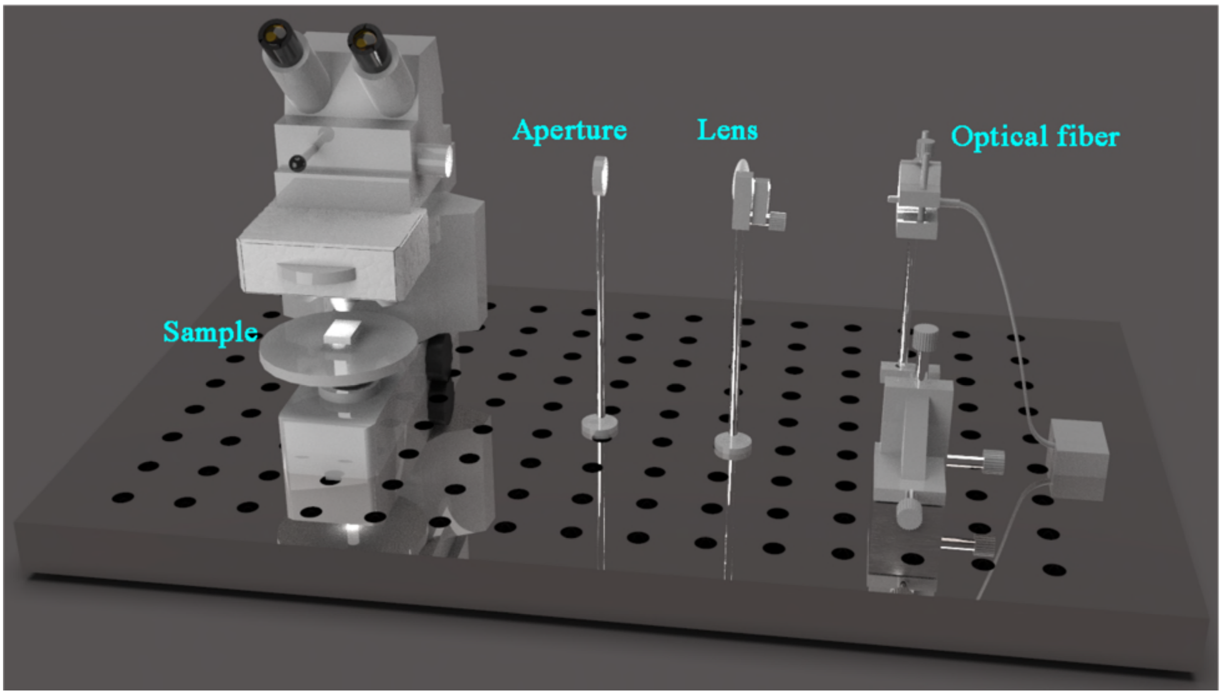

Supplementary Figure 11. Schematic illustration of the home-built setup of the optical microscope (ZEISS, Axio Scope AI). The 50× objectives were used to illuminate the sample.

Supplementary Note 12:Optical and scanning electron micrographs of peacock with large size

In the main manuscript, we have discussed the image of peacock. Here we show the corresponding SEM image, the original image, and the image in the air. As shown in Supplementary Figure 12, while some pixels have very small sizes, structural color can still be realized. Similar to the other images, the colors in DMSO are more distinct and have a dark background.

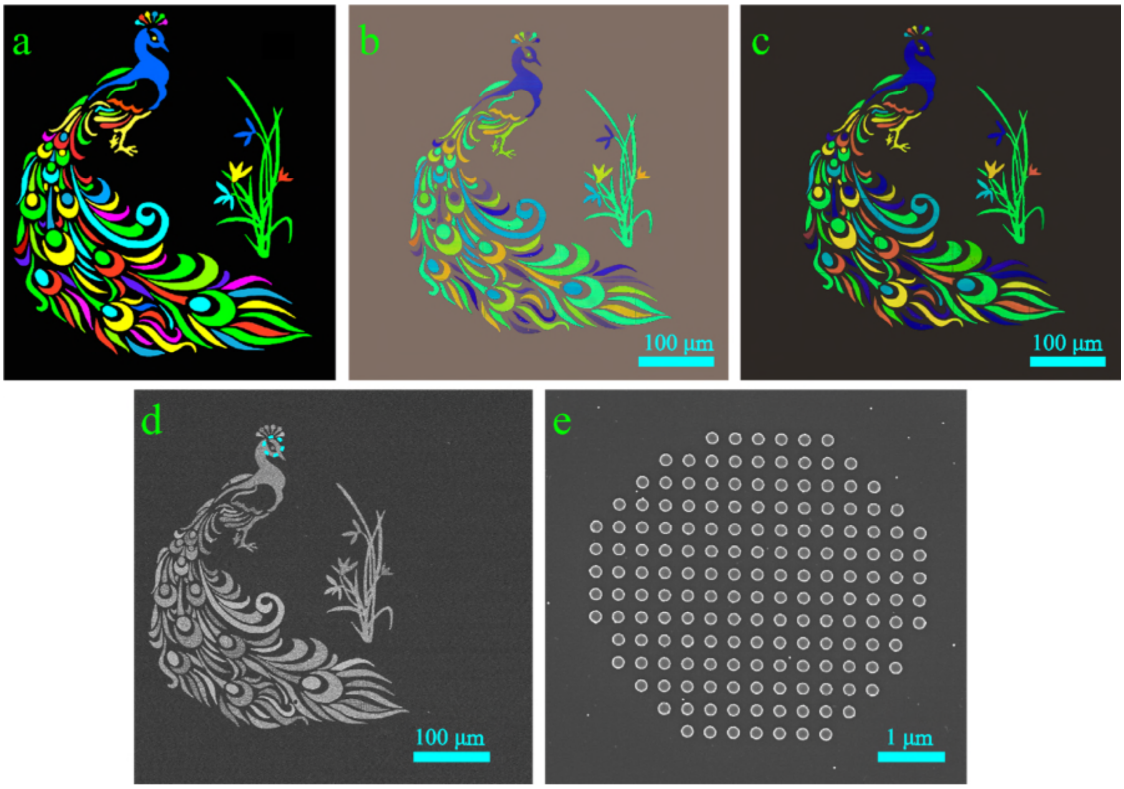

Supplementary Figure 12. (a) The original design peacock pattern**.** (b) and (c) are the corresponding bright-field microscope images in air and DMSO solutions, respectively. (d) The top-view SEM image of a peacock with an orchid. The scale bar is 100 μm. (e) The magnifying SEM image in in (d) surrounded with the cyan circle. The scale bars are shown in the SEM image.

Supplementary Note 13: The calculate method of the gamut of structural color

In this work, Finite-difference time-domain (FDTD) simulations were used with the Lumerical FDTD Solutions software. Periodic boundary conditions were set and the incident optical field is linear polarization with a normal plane wave. The unit structure was composed of Si nanodisk on top of a sapphire substrate, with the lossless refractive index of substrate n= 1.76. The optical constant of crystalline silicon was taken from the FDTD by Palik.

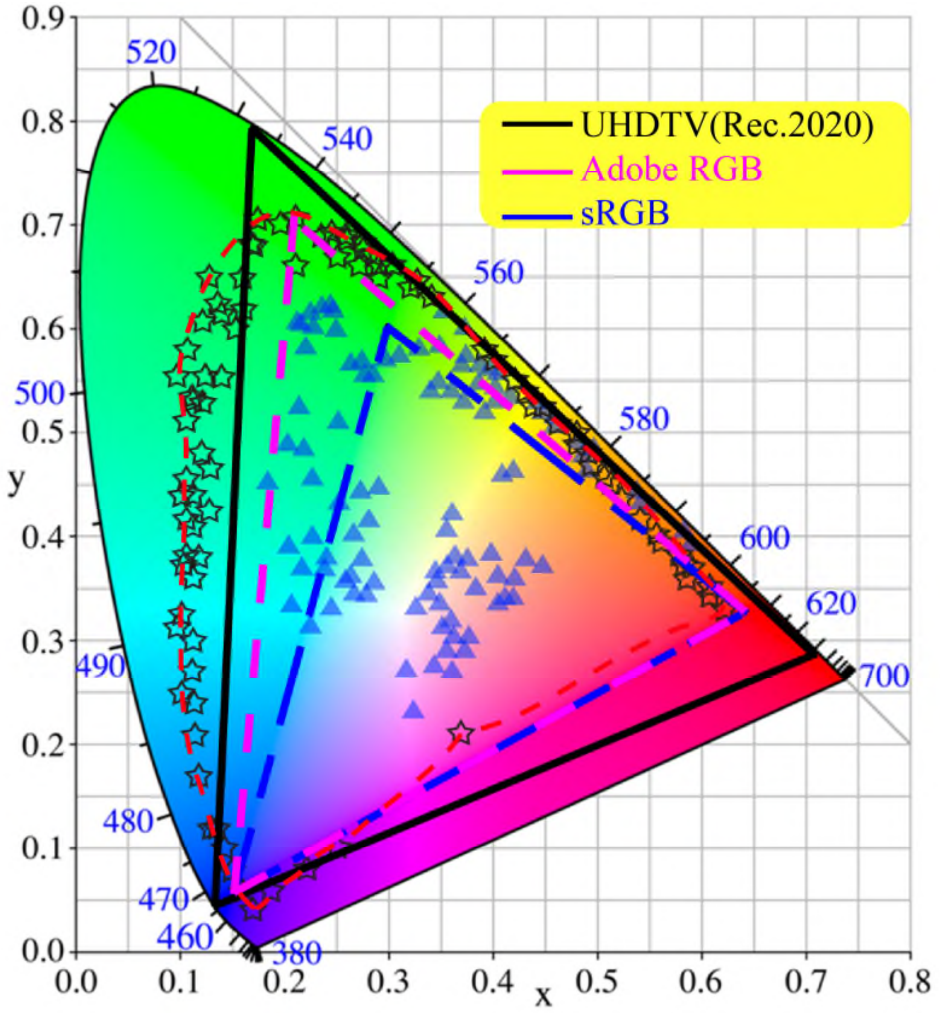

Supplementary Figure 13. The gamut of the experiment silicon colors in the CIE chromaticity diagram plotted by the red dashed line. The gamut of the sRGB in the CIE is calculated to be 136017. The gamut of the Adobe RGB in the CIE is calculated to be 182304. The gamut of the (UHDTV) Rec.2020 in the CIE is calculated to be 254178. The gamut of the measured color palette is 247300, which is 181.8% of the sRGB

areal coverage. 135.6% of Adobe RGB and 97.2% of Rec.2020. The gamut measured by the Photoshop software.

Supplementary Note 14:The fabrication progress of the silicon nanodisk with ZEP photoresist

In order to save fabrication time, we also perform fabrication of silicon metasurface using ZEP520 photoresist as etching mask since ZEP520 typically has a 1:1 dry plasma etch selectivity relative to Si. We first spin-coat ZEP520 electron beam resist with thickness of 350 nm on top of the 100 nm silicon on sapphire substrate. After baking at 180 °C for 1 hour, Electron beam lithography (EBL) method is used to pattern the nanostructure on the resist. The reverse pattern of the nanodisks structure is wrote on the ZEP resist. After that, the resist is developed in the ND510 solution for 60 s at 0 °C. Since ZEP520 is a positive tone resist, nanodisks structure will remain in the resist and acts as the soft mask for silicon etching. Then the silicon structure is obtained by anisotropy etching with the reactive ion etching (RIE) process using SF_6 as etching gas and CHF_3 as protect gas at a RF power of 300 W. After immersing the sample in acetone for 8 h, the remaining ZEP is removed and the Si metasurfaces are formed.

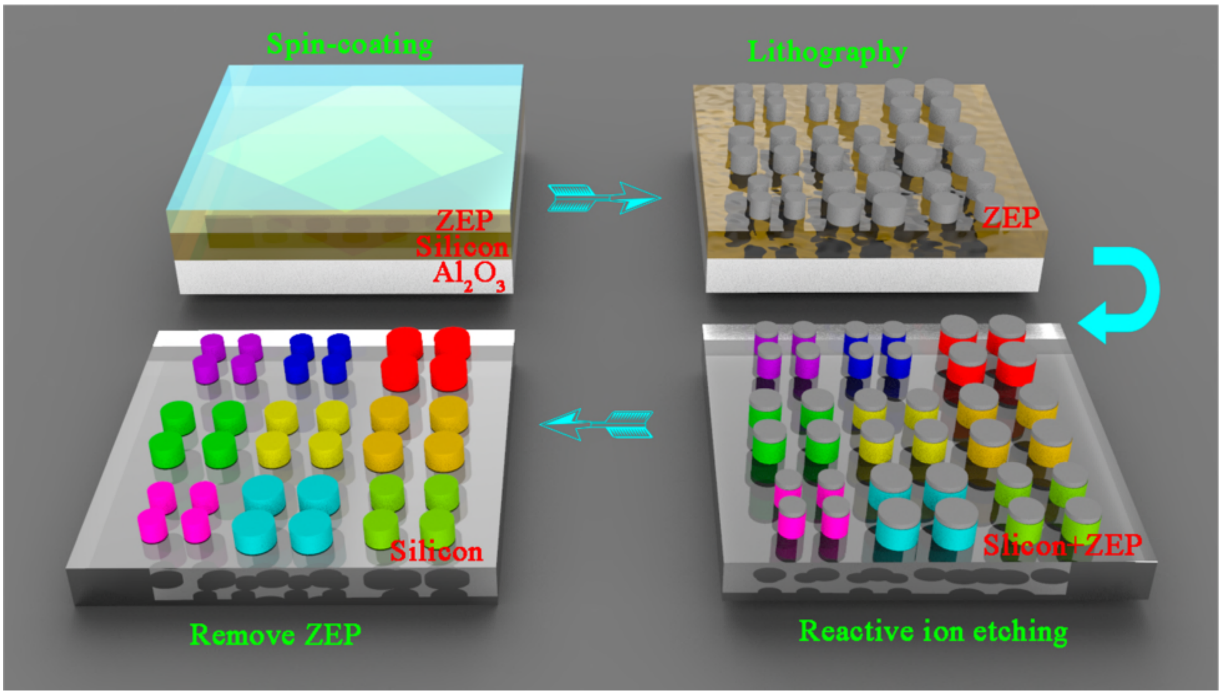

Supplementary Figure 14. The schematic of the fabrication process for silicon nanodisk structural color with ZEP photoresist.

Supplementary Note 15: The gamut of silicon nanodisk fabricated by the ZEP method

After we use the ZEP method (shown in Supplementary Note 14) to fabricate the structural color, the fabrication time can be reduced. The experimentally demonstrated gamut has been increased to a value around 153% of sRGB, 115% of Adobe RGB, and 83% of Rec.2020 shown in Supplementary Figure 15(a). We also fabricate the 128 colors palette. Supplementary Figure 15(b) and (c) represent the sample in Air and DMSO respectively. Same with the PMMA method, after adding the refractive index matching layer, the color becomes pure and more saturation. The gamut of color also become wider. However, the overall performances of Si metasurfaces fabricated with ZEP are still worse than the results in Fig. 3(d) in the main text. Therefore, we keep the lift-off process and use the Cr hard mask in the experiments.

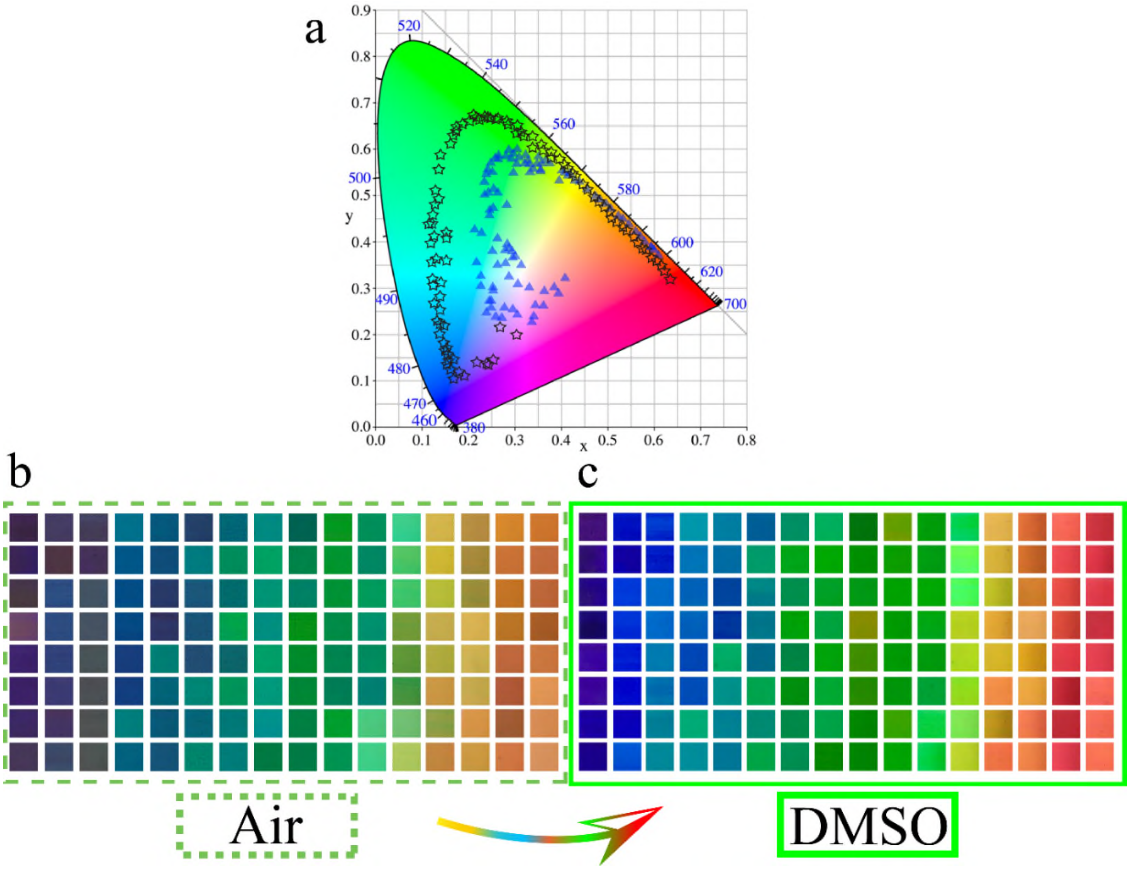

Supplementary Figure 15. The experimentally recorded structural color. (a) The experimentally recorded color palettes of 128 Si metasurfaces in the air (triangles) and in DMSO (stars), respectively. (b) and (c) are the experimentally recorded color palettes of 128 Si metasurfaces in the air and in DMSO, respectively.

Supplementary Note 16: Calculate method of the CIE 1931 coordinate for the reflection case

The reflective cases are very similar to the emissive case, with a few differences. The spectral radiance is replaced by the spectral reflectance $R(\lambda)$ of the object being measured, multiplied by the spectral power distribution of the illuminant $S(\lambda)$.

$$\mathrm{X} = k\int_{\lambda} S(\lambda)R(\lambda)\overline{x}(\lambda)d\lambda$$

$$\mathrm{Y} = k\int_{\lambda} S(\lambda)R(\lambda)\overline{y}(\lambda)d\lambda \qquad (1)$$

$$\mathrm{Z} = k\int_{\lambda} S(\lambda)R(\lambda)\overline{z}(\lambda)d\lambda$$

$\overline{x}(\lambda)$, $\overline{y}(\lambda)$, $\overline{z}(\lambda)$ in this system are called the color-matching functions, where k constant is chosen for a normalized value Y = 100.

By means of the XYZ tristimulus values, we can define the chromaticity coordinates *x y z*. that is:

$$x = \frac{X}{X+Y+Z}$$

$$y = \frac{Y}{X+Y+Z} \qquad (2)$$

In this paper, we use the FDTD Solutions software to simulate the optical reflectance spectrum from the silicon nanodisks with the different diameter and period. Then the simulated reflectance spectrum for each color pixel is converted to *xy* coordinate in the CIE 1931 chromaticity diagram.

Supplementary Note 17: The further optimization of structural color

In the main text, we have shown that the combination of refractive index matching layer and Si metasurface is a good way to approach the ultimate limits of structural color. However, it is important to note that the results in the main text are not extremely optimized. There are still rooms to improve the performances. To illustrate this statement, we perform more numerical calculations and experiments to optimize the reflection of the silicon metasurface at shorter wavelengths. As shown in Supplementary Figure 16(a), through optimizing five parameters in simulation, the highest reflection of a metasurface with diameter/period of 120/200 nm can reach as high as 65% at a wavelength around 500 nm (for green color) when the sample is placed in air. Similarly, the highest reflectance of Si metasurface with diameter/period of 80/140 nm can reach 53% at a wavelength around 430 nm (for blue color). After adding the refractive index matching layer, the highest reflectance peak at 500 nm and 430 nm only slightly deceases to 62% and 47% (see Supplementary Figure 16(b)), demonstrating that the refractive index matching layer does not reduce the reflectance very much. The above simulations have been further confirmed with the corresponding experiments. The peak values in Supplementary Figure 16(d) are still around 60% and 45%, consistent with the numerical simulation very well. We have to admit that the size parameters and the geometry are not ultimately optimized. By carefully changing the geometry and sizes, the color performances in Fig. 1 of main text can be further improved.

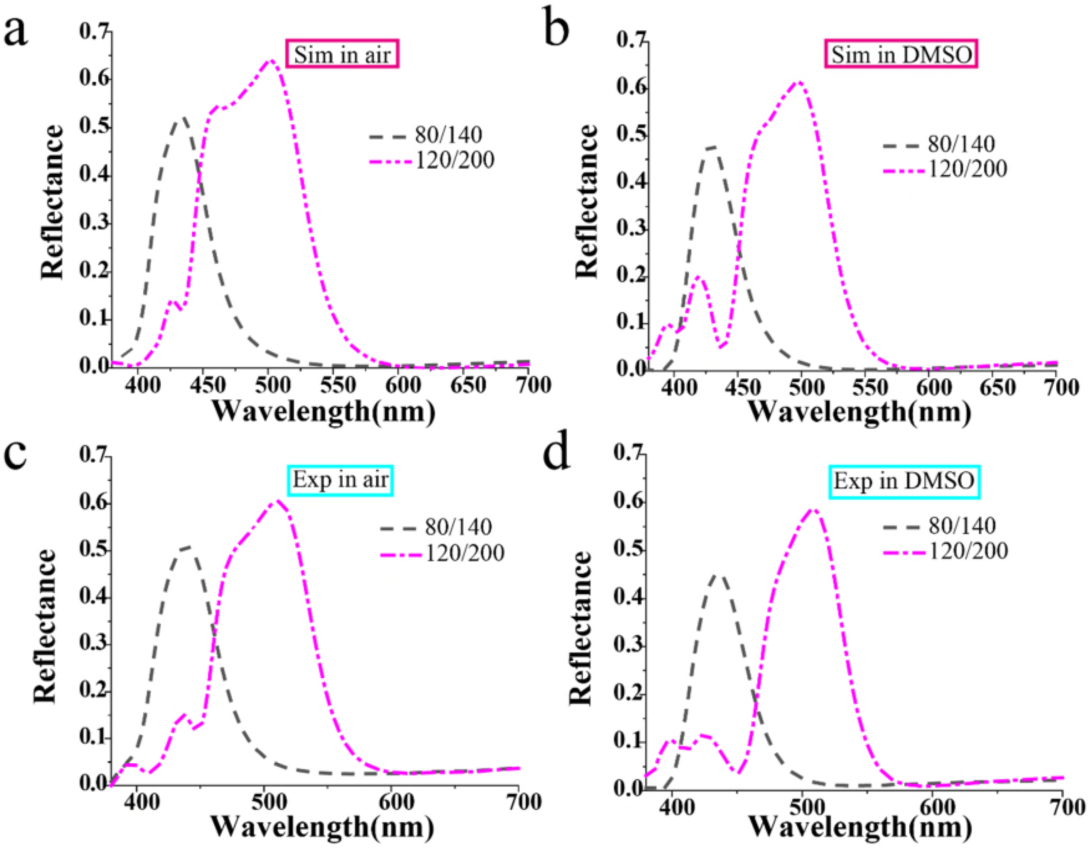

Supplementary Figure 16. The optimization of structural color at shorter wavelength. (a) and (b) are the calculated reflection spectra of five color at wavelength <500 nm with different lattice sizes in air and in DMSO, respectively. The insets are the corresponding calculated structural color. (c) and (d) are the measured reflection spectra of three samples in air and with DMSO, respectively. The diameter/period (nm) parameters are labelled in the figures.

Supplementary Note 18:The multipolar decomposition analysis

In the main text, we have observed numerically and experimentally that the reflection background is reduced and the reflection peak is narrowed. Here we employ the multipolar decomposition analysis to study the underlying mechanism for the improved color performances. In the calculation of multipolar decomposition, we expand the total scattering power into electric dipole (ED), magnetic dipole (MD), electric quadrupole (EQ) and magnetic quadrupole (MQ). In the calculation of multipolar decomposition. In the case of harmonic excitation $exp(i\omega t)$, the power of scattering cross section can be expressed as:

$$\mathrm{I} = \frac{2\omega^4}{3c^3}|P|^2 + \frac{2\omega^4}{3c^3}|M|^2 + \frac{\omega^6}{5c^5}Q_{\alpha\beta}Q_{\alpha\beta} + \frac{\omega^6}{20c^5}M_{\alpha\beta}M_{\alpha\beta} \quad (1)$$

The electric dipole moment is defined as $P = \frac{1}{i\omega}\int j\, d^3r.$ (2)

The magnetic dipole moment is $M = \frac{1}{2c}\int(r \times j)\, d^3r.$ (3)

The electric quadrupole moment is $Q_{\alpha\beta} = \frac{1}{i\omega}\int \left[r_\alpha r_\beta + r_\beta r_\alpha - \frac{2}{3}(r \cdot j)\right] d^3r.$ (4)

And the magnetic quadrupole moment is $M_{\alpha\beta} = \frac{1}{3c}\int\left[(r \times j)_\alpha r_\beta + (r \times j)_\beta r_\alpha\right] d^3r$. (5)

Here j represent current density and c is light speed.

To illustrate the universal of the mechanism, we take three Si metasurfaces with blue, green, and red colors as examples to demonstrate the effects of refractive index matching layer. All the results are shown in Supplementary Figure 17-Supplementary Figure 19. When the Si metasurfaces are placed in air, the reflection spectra show three relatively broad reflection peaks can be seen in Supplementary Figure 17(a), Supplementary Figure 18(a) and Supplementary Figure 19(a). The corresponding multipolar decomposition results in Supplementary Figure 17(b), Supplementary Figure 18(b) and Supplementary Figure 19(b) show that these peaks are dominated by ED resonance and MD resonance. The phase difference between ED and MD resonances are between 0-π/2 in Supplementary Figure 17(c), Supplementary Figure 18(c), and Supplementary Figure 19(c). Consequently, the intensities of ED and MD at the other wavelengths are constructively interact one another and generate a relatively high reflection background.

When the refractive index matching layer is applied, the multipolar decomposition results in Supplementary Figure 17(d), Supplementary Figure 18(d) and Supplementary Figure 19(d) show that the intensities of ED resonance are greatly enhanced and shifted to longer wavelength.

The MD resonances are mostly kept at their original positions and values. Meanwhile, the phase differences between ED and MD resonances increases to π/2-π (see Supplementary Figure 17(f), Supplementary Figure 18(f), and Supplementary Figure 19(f)). The former one makes ED and MD overlap one another largely and narrow the overall linewidth, whereas the latter one introduces destructive interference and reduces the background reflection. Therefore, the reflection peaks in Supplementary Figure 17(b), Supplementary Figure 18(b) and Supplementary Figure 19(b) become much narrower and the background reflection is reduced to ~ 0. These two effects are essential for the improvements of color performances. The different responses of ED and MD resonances lie in their electromagnetic field distributions. As shown in Fig. 2 of the main text, the ED resonance has larger portion of electromagnetic field that is localized outside the Si nanodisk. As a comparison, the MD resonance is well confined within the nanodisk. Consequently, the former one shows more significant change when the refractive index of surrounding medium is changed.

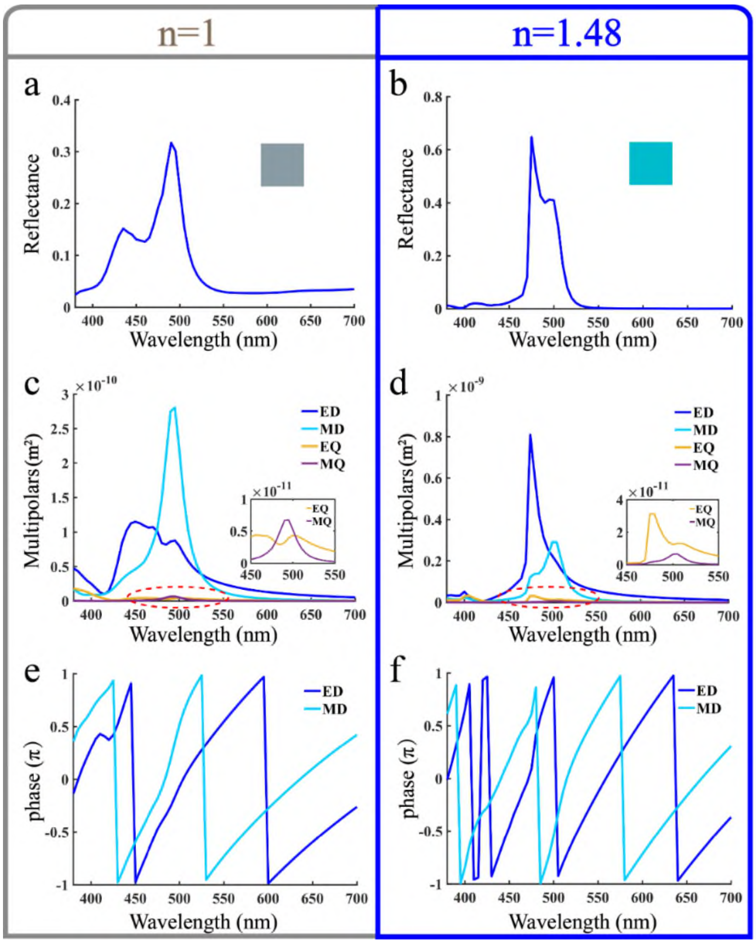

Supplementary Figure 17. Optical characteristics of blue-color silicon nanostructures with n=1(left column) and n=1.48 (right column). (a) and (b) Simulated reflectance spectra of silicon nanodisks (diameter/period 100/270 nm). The insets are the corresponding structural color in simulation. (c) and (d) Multipolar decomposition of scattering cross-sections for ED, MD, EQ, and MQ. The insets show the magnification of decomposition at wavelength ranging from 450 nm to 550 nm. (e) and (f) Phase distribution of electric dipole and magnetic dipole retrieved from the multipolar decomposition.

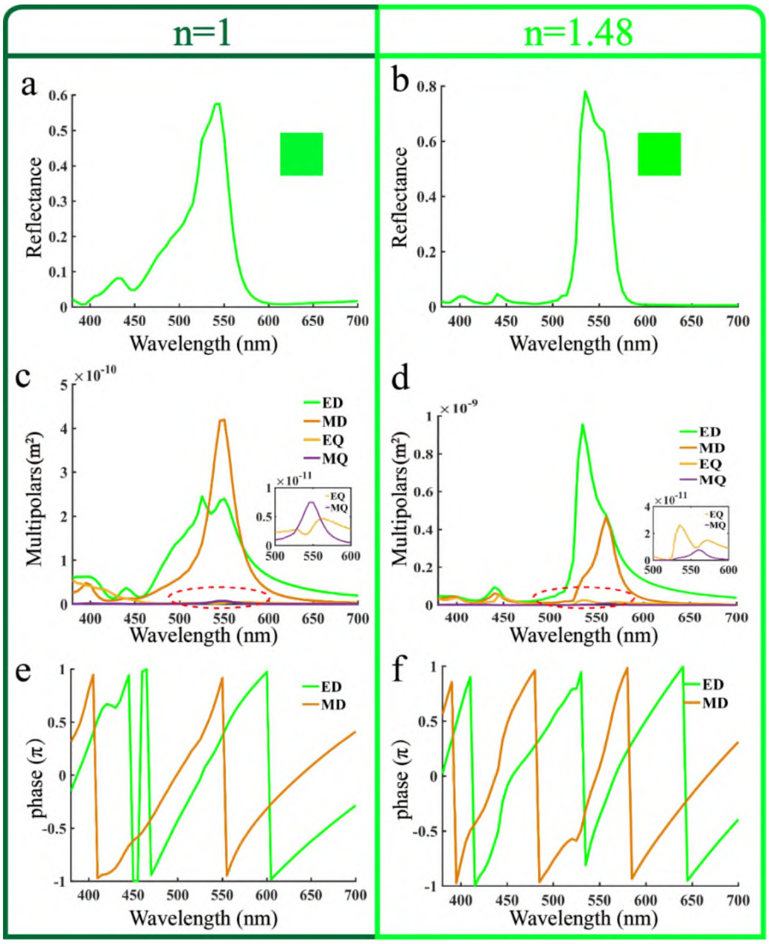

Supplementary Figure 18. Optical characteristics of green-color silicon nanostructures with n=1(left column) and n=1.48 (right column). (a) and (b) Simulated reflectance spectra of silicon nanodisks (diameter/period 130/300 nm) by COMSOL Multiphysics. The insets are the corresponding structural color in simulation. (c) and (d) Multipolar decomposition of scattering cross-sections for ED, MD, EQ, and MQ. The insets show the magnification of decomposition at wavelength ranging from 500 nm to 600 nm. (e) and (f) Phase distribution of electric dipole and magnetic dipole retrieved from the multipolar decomposition.

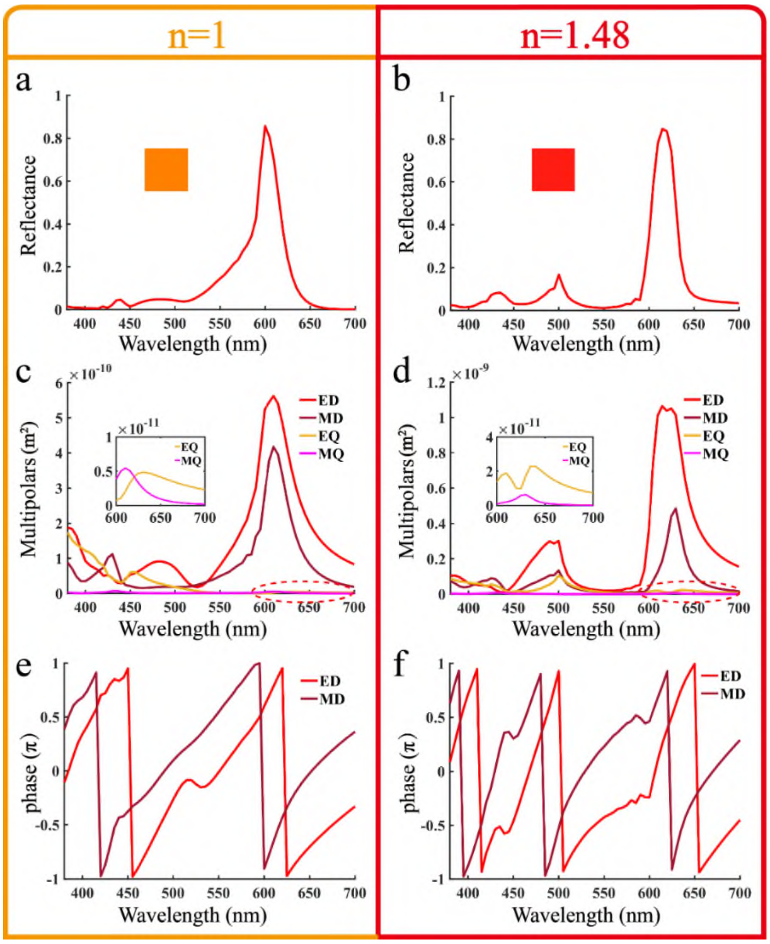

Supplementary Figure 19. Optical characteristics of red-color silicon nanostructures with n=1(left column) and n=1.48 (right column). (a) and (b) Simulated reflectance spectra of silicon nanodisks (diameter/period 170/340 nm). The insets are the corresponding structural color in simulation. (c) and (d) Multipolar decomposition of scattering cross-sections for ED, MD, EQ, and MQ. The insets show the magnification of decomposition at wavelength ranging from 600 nm to 700 nm. (e) and (f) Phase distribution of electric dipole and magnetic dipole retrieved from the multipolar decomposition.

Supplementary Note 19:The angular dependence

For structural color, the angular dependence and polarization dependence are also important since they are mainly used for a wide view angle and under natural white light. In the main text, we have mentioned that the structural color with or without refractive index matching layer are quite robust to the view angle and doesn't require specific polarization. Here we take one Si metasurface as an example and show the numerical and experimental details. The diameter of Si nanodisk and the lattice size are 90 nm and 200 nm, respectively. Supplementary Figure 20 shows the angular dependent reflection spectrum of the Si metasurface. When the Si metasurface is placed in air, as shown in Supplementary Figure 20(a), the reflection spectrum keeps at the same wavelength with the increase of incident angle from 0 to 60 degree. The p-polarization in Supplementary Figure 20(e) is slightly different and the intensity decreases at larger angle. In case of non-polarized white light, the overall structural color shall be well preserved.

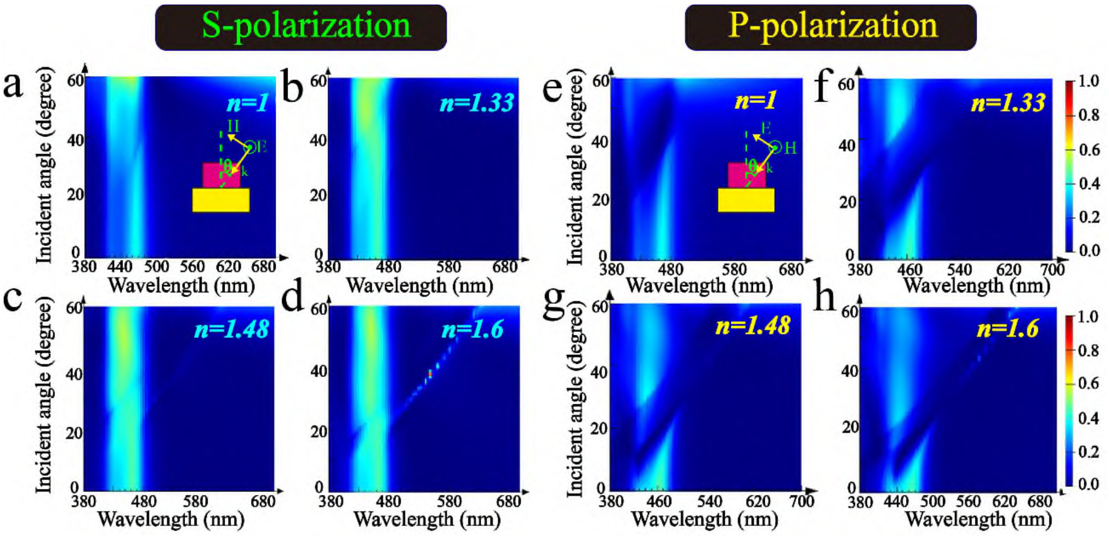

Supplementary Figure 20. The angular dependence of structural color. (a)-(d) and (e)-(h) are Simulated angular dispersion of the reflection spectrum of diameter/period (nm) 90/200 nm nanodisks with the S polarization and P polarization, respectively. (a)-(d) and (e)-(h) are both with refractive index matching layer, n=1, 1.33, 1.48, and 1.6, respectively. The inset shows the angle θ of the incident light.

Supplementary Figure 20(b)-Supplementary Figure 20(d) show the angular dependent reflection spectrum of the same Si metasurface with different refractive index matching layer. With the refractive index matching layer, the reflection peak shifts to the long wavelengths. However, their angular dependences are still the same. With the increase of refractive index from

1 (Supplementary Figure 20(a) and Supplementary Figure 20(e)) to 1.33 (Supplementary Figure 20(b) and Supplementary Figure 20(f)), 1.48 (Supplementary Figure 20(c) and Supplementary Figure 20(g)), and 1.6 (Supplementary Figure 20(d) and Supplementary Figure 20(h)), both of the s-polarized incident light and p-polarized incident light are kept at the same wavelengths. Similar angular dependences have also been observed in other structural color. Therefore, similar to the previously reported dielectric metasurfaces, the structural color are also quite robust to the incident angle.

With the above simulation, we take one Si metasurface as an example to measure its reflection spectrum as a function of incident angle. Here the diameter of nanodisk and the lattice size are 150 nm and 240 nm. After covering the sample with DMSO (n = 1.48), an Apple green has been achieved as shown in the main text. Supplementary Figure 21(a) shows the reflection spectrum of the Si metasurface as a function of incident angle. With the increase of incident angle from 0° to 43°, the main reflection peak is well kept at ~ 550 nm. All of these spectral responses are consistent with the numerical calculations in Supplementary Figure 21(b) very well. The insets in Supplementary Figure 21(a) are the experimentally recorded un-polarized microscope images at different angle. It is easy to see that the Apple green color is well preserved with the increase of incident angle. This observation also matches the numerical results (insets in Supplementary Figure 21(b)) very well.

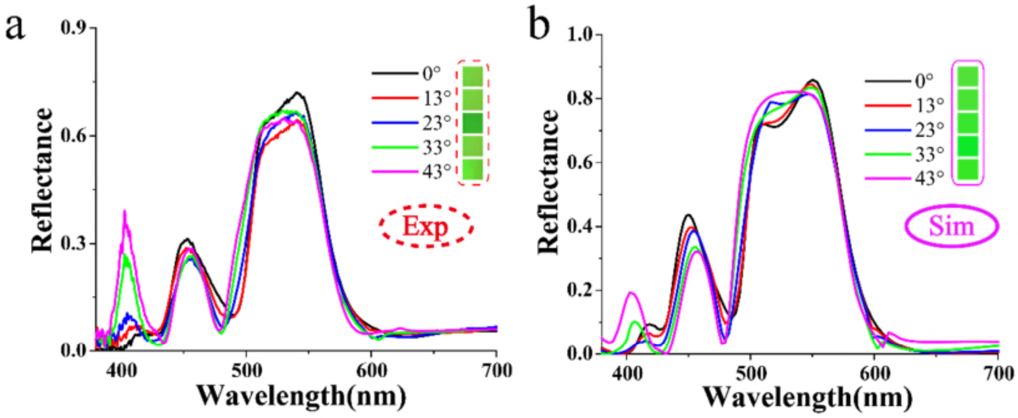

Supplementary Figure 21. (a) The measured reflectance of diameter/period (nm) 150/240 nm with five incident angle 0°, 13°, 23°, 33°, and 43°. (b) The simulated reflectance of diameter/period (nm) 150/240 nm with five incident angle 0°, 13°, 23°,33°, and 43°. The inset color images in (a) and (b) are the experimentally recorded structural color and the corresponding numerical calculations.

We have also studied the dependence of color performance of the pixels on the viewing angle. We simulate the scattering cross section of 2 × 2 pixels for three primary colors. The results are shown in Supplementary Figure 22. The diameter/period (nm) for these three primary colors are 80/140 nm, 120/240 nm, and 170/320 nm, respectively and the viewing angles are 0° and 60°. As shown in Supplementary Figure 22, both of the peak position and the intensity of reflectance for 2 × 2 pixels almost keep the same when the for the angles of 0° and 60°, indicating the viewing angle independent characteristic both for the period structure in color palettes and the small pixels. The inset color images are also calculated from the spectrum, all these three primary colors keep the same, confirming the viewing angle independent characteristic. Supplementary Figure 22(b) (d) (f) further demonstrate the viewing angel-independent characteristic of the 2 × 2 pixels in DMSO with n=1.48. In the same way, both the position of peak and the intensity of reflectance keep almost unchanged with the increasing viewing angel. The refractive index matching layer will not influence the effect of view angle and the viewing angle independent property of the Si nanodisk is also well attained.

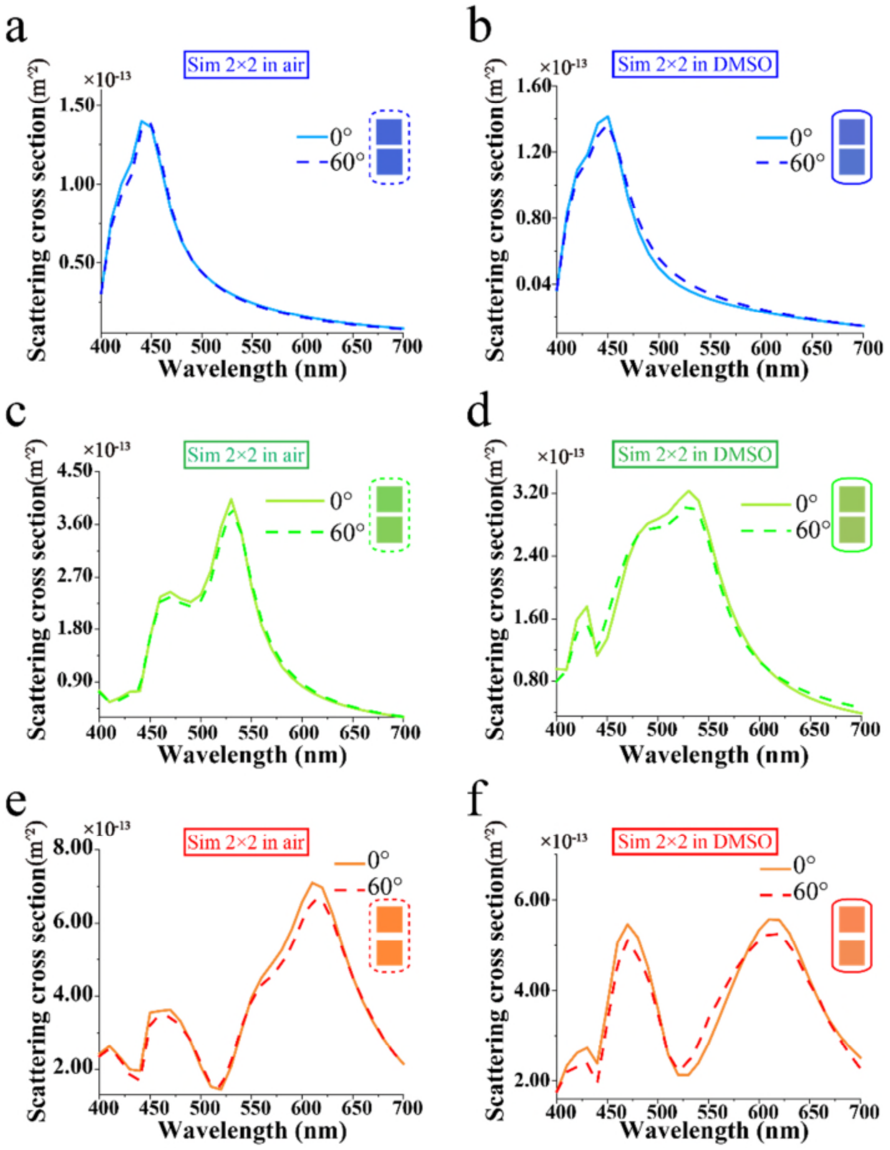

Supplementary Figure 22. Angle-dependence characteristic for the 2 × 2 pixels. (a) (c) (e)and (b) (d) (f)The simulation scattering cross section of diameter/period (nm) 80/140 nm, 120/240 nm, and 170/320 nm with 2 × 2 nanodisks in air and DMSO, respectively. The incident angel is 0° and 60°, respectively. The inset images are corresponding color calculated from spectrum.

Supplementary Note 20: The effect of absorption in Si

To observe the effect of absorption in Si, we simulate the absorption inside the silicon nanodisks for three primary colors when the metasurfaces are placed in air or in the refractive index matching layer. By adding a refractive index matching layer with n = 1.48 onto the surface of the nanodisks, the match of refractive index to the substrate and the Kerker condition will result in the suppression of reflection background. This will induce the reduction of absorption outside the resonant wavelength. At the resonant wavelength, due to the increased scattering cross sections of electric dipole resonances (see Supplementary Figure 17-Supplementary Figure 19), the absorption of silicon shall slightly increase. This analysis can be clearly seen in Supplementary Figure 23 below. In most of cases, the absorptions at the resonant wavelengths are slightly increased by the refractive index matching layer. This is also consistent with the results in main text, where the reflection peak values in DMSO are slightly lower than the ones in air.

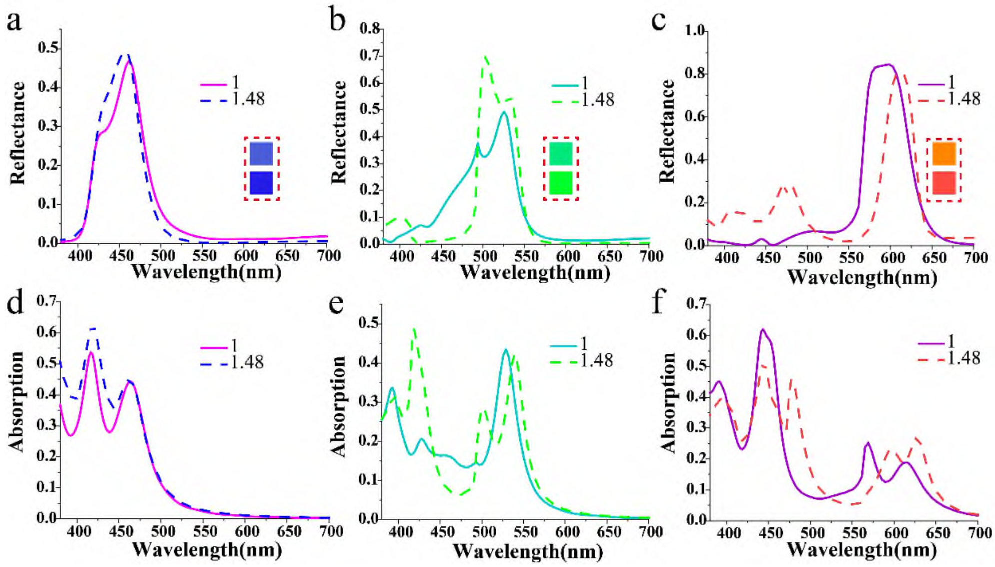

Supplementary Figure 23. (a) (b) (c) The reflection of diameter/period 90/180 nm, 120/280 nm and, 180/320 with n=1 and 1.48. (d) (e) (f) The absorption of diameter/period 90/180 nm, 120/280 nm and, 180/320 nm with n=1 and 1.48.

Supplementary Note 21: The package with solid-state materials

In the main text, we have shown that the refractive index matching layer can be either the solvent such as DMSO or the solid-state materials such as polymethyl methacrylate (PMMA). The color performances with solvent and the solid-state material are almost identical. Here we show the details of the packaging with PMMA. As demonstrated in Supplementary Figure 24, the Si metasurface can be placed into a plastic bottle cap (see Supplementary Figure 24(a)) and immerse with the 1.5 mL PMMA solution. After standing still in air for about 2 hours, the PMMA liquid will volatilize and then totally become solid state. The solid layer attaches to Si metasurface very closely and the whole sample can be simply tear off (see Supplementary Figure 24(b)). Similar process holds true for epoxy-based negative photoresist (SU-8) as well.

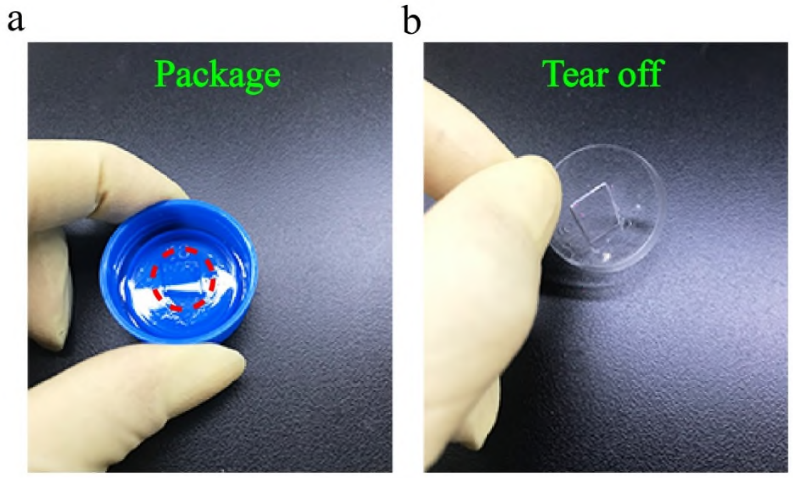

Supplementary Figure 24. The package state of the Si metasurface. (a) The Si metasurfaces package with the PMMA solid layer after immersed in PMMA liquid 2 hours. The sample is surrounded by the red dotted line. (b) The refractive index matching layer can be teared off without damage the Si metasurface.

It is important to note that the structural color can be well preserved after replacing the DMSO with PMMA or SU8. Supplementary Figure 25 shows the experimental results. We can see that the yellow, green and blue colors in air becomes distinct red, green, and blue colors (see Supplementary Figure 25(b)). When the DMSO is replaced with PMMA, the structural color in Supplementary Figure 24(c) are well kept and almost the same as Supplementary Figure 25(b). This kind of packaging can be repeated for many rounds. As shown in Supplementary Figure 25(d)-Supplementary Figure 25(l), the packing process has been repeated for another 9 rounds without obvious degradation in color impressions. This clearly shows the robustness of the packaging process as well as the durability of Si metasurface.

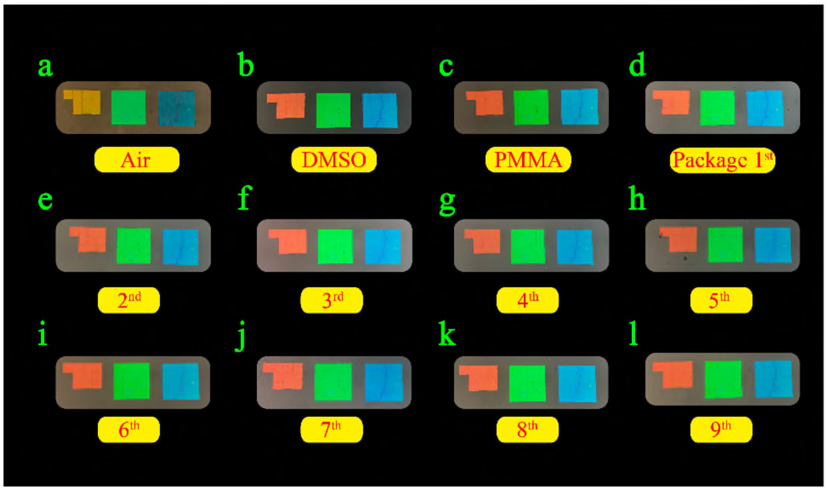

Supplementary Figure 25. The repetitive experiments for PMMA packaging experiment.

In order to further demonstrate this method can be used to realize all solid structural color, we package the "phoenix" pattern and "福" with fish and plum blossom pattern using the same method. Supplementary Figure 26(a) shows the colors of the three patterns in the air condition, and the dark cyan, apple green, bitter lime, and carrot orange are presented. After packaged with the PMMA solid refractive index matching layer, the color change to cyan, bright green, yellow and red shown in Supplementary Figure 26(b). At the same time, all the colors become more distinct, proving the obvious improvement of color performance.

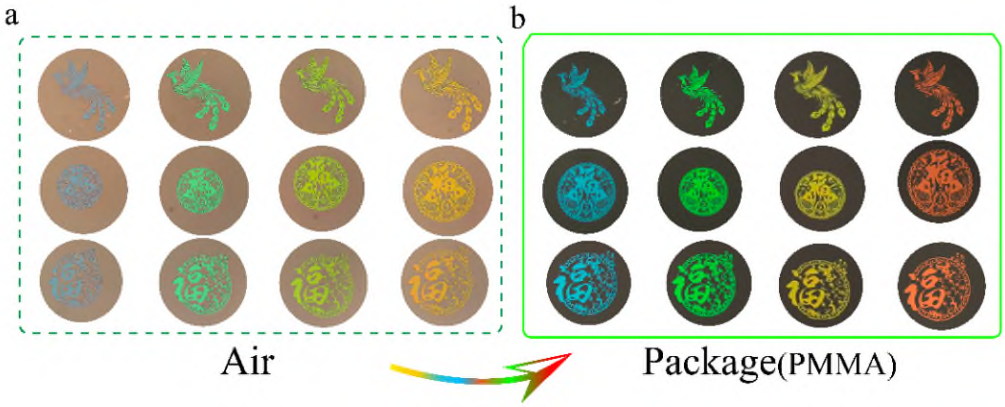

Supplementary Figure 26. The comparison between the four pure color of "phoenix", "福" with two fish and plum blossom in air and package state. (a) The experimentally recorded bright-field microscope images of "phoenix" in air with different pitch sizes. (b) is the experimentally recorded corresponding images of "phoenix" in PMMA package state. The diameter/period of four color are 120/240 nm, 150/240 nm, 160/300 nm, and 170/320 nm, respectively.

Similar improvements also hold true for the peacock image in the main text. By replacing the DMSO with PMMA as refractive index matching layer, it is easy to see that the color impression of the peacock is almost the same as the previous one.

To further demonstrate the package method, seven colors in visible spectrum are shown in Supplementary Figure 27. Supplementary Figure 27(a) shows the simulated reflection spectrum of three samples at wavelength <500 nm with different lattice sizes in DMSO. The reflectance in short wavelength range can beyond 60% after optimizing. Supplementary Figure 27(c) shows the measured reflection spectrum of three samples with the solid state PMMA (after package). The reflectance still keeps very high. The inset colors are the experimentally recorded corresponding color palettes with the PMMA, which are vivid and pure, and almost the same with the simulated colors in DMSO. Supplementary Figure 27(b) shows the simulated reflection spectrum of four samples at wavelength >500 nm in DMSO, and Supplementary Figure 27(d) shows the measured reflection spectrum of relevant samples at wavelength >500 nm with solid PMMA. The insets are the corresponding structural color in experiment. In the same way, the simulation and experiment results are matched very well, which further demonstrates the success of the package method.

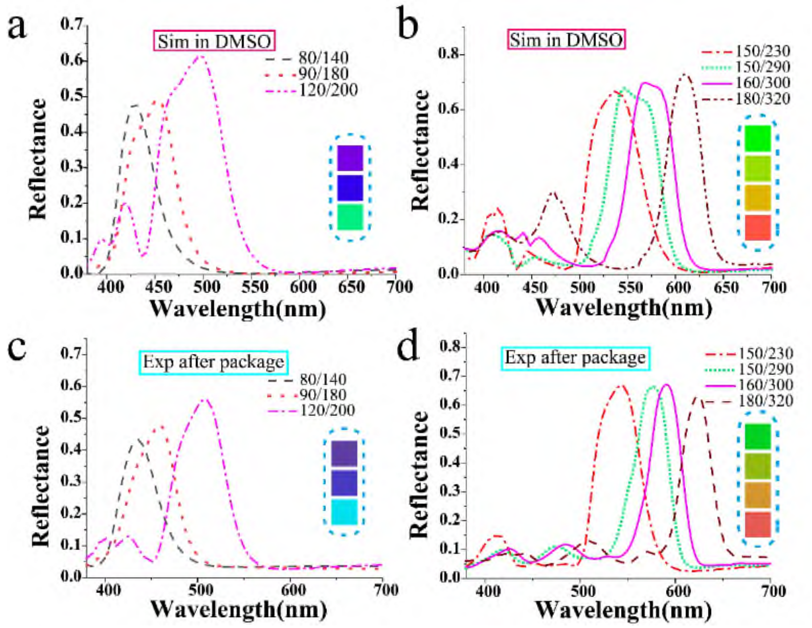

Supplementary Figure 27. The comparisons of simulated and measured reflectance spectrum/ corresponding color between DMSO solution and PMMA (after package). (a) The simulated reflection spectrum of three samples at wavelength <500 nm with different lattice sizes in DMSO. (b) The simulated

reflection spectrum of four samples at wavelength >500 nm with different lattice sizes in DMSO. (c) The measured reflectance spectrum with PMMA package layer of three samples at wavelength <500 nm. (d) The measured reflectance spectrum with PMMA package layer of four samples at wavelength >500 nm. The insets are the corresponding structural color in simulation and experiment.
